# Supplementary material for: Regulating cleavage activity and enabling microRNA detection with split sgRNA in Cas12b
Source: Nat Commun. 2025 Jul 10;16:6392. doi: 10.1038/s41467-025-61748-4 (PMC12246129; doi:10.1038/s41467-025-61748-4)
Supplement: Supplementary file 1 — Supplementary Information [file 41467_2025_61748_MOESM1_ESM.pdf]

Supplementary Information

Regulating Cleavage Activity and Enabling MicroRNA Detection with Split sgRNA in Cas12b

Jiaqi Wang[1,†], Xiaofang Ye[1,†], Yuanfang Liu[1,†], Wentao Li[2], Xue Zhang[1], Wei Zhang[1], Changqing Yi[3], Chaoxing Liu[1,*]

[1] Guangdong Provincial Key Laboratory of Digestive Cancer Research, Digestive Diseases Center, Scientific Research Center, The Seventh Affiliated Hospital of Sun Yat-sen University, Shenzhen, Guangdong, 518107, P. R. China.

[2] Department of Clinical Laboratory, The Seventh Affiliated Hospital of Sun Yat-sen University, Shenzhen, Guangdong, 518107, P. R. China.

[3] Guangdong Provincial Key Laboratory of Sensor Technology and Biomedical Instrument, School of Biomedical Engineering, Shenzhen Campus of Sun Yat-Sen University, Shenzhen, Guangdong, 518107, P. R. China.

[†] These authors contributed equally to this work.

* To whom correspondence should be addressed. Tel: +1-951-888-1287, https://orcid.org/0000-0003-3077-9660; Email: liuchx69@mail.sysu.edu.cn

ODN sequences used in this study

Supplementary Table 1. Sequences of DNA or RNA oligonucleotides used in this work.

Name	Sequence (from 5' to 3')
EBV Spacer	CAAACUCAUAUAUUUGCUGA
Spacer-3′-Cy5	CAAACUCAUAUAUUUGCUGA/**Cy5/**
EBV crRNA	CGAGCGAUCUGAGAAGUGGCACCAAACUCAUAUAUUUGCUGA
crRNA-3′-Cy5	CGAGCGAUCUGAGAAGUGGCACCAAACUCAUAUAUUUGCUGA/**Cy5/**
DR	AUCUGAGAAGUGGCAC
DR-FAM	**/56-FAM/**AUCUGAGAAGUGGCAC
tracrRNA	GUCUAGAGGACAGAAUUUUUCAACGGGUGUGCCAAUGGCCACUUUCCAGGU GGCAAAGCCCGUUGAGCUUCUCAA
tracrDR	GUCUAGAGGACAGAAUUUUUCAACGGGUGUGCCAAUGGCCACUUUCCAGGU GGCAAAGCCCGUUGAGCUUCUCAAAUCUGAGAAGUGGCAC
EBV sgRNA	GUCUAGAGGACAGAAUUUUUCAACGGGUGUGCCAAUGGCCACUUUCCAGGU GGCAAAGCCCGUUGAGCUUCUCAAAUCUGAGAAGUGGCACCAAACUCAUAU AUUUGCUGA
sgRNA-3′-Cy5	GUCUAGAGGACAGAAUUUUUCAACGGGUGUGCCAAUGGCCACUUUCCAGGU GGCAAAGCCCGUUGAGCUUCUCAAAUCUGAGAAGUGGCACCAAACUCAUAU AUUUGCUGA/**Cy5/**
HCV Spacer	GGCGUGCCCCCGCAAGACUG
MPXV Spacer	GUAUAUAAGUUGUACGGCUA
SPET9 Spacer-1	AGCGGGGCCUGAGAGCCGCC
SPET9 Spacer-2	AGCGGGGCCUGAGAGCUGCC
SPET9 Spacer-3	AGCGGGGCCUGAGAGCUGCU
SPET9 Spacer-4	AGUGGGGCCUGAGAGCUGCU
SPET9 Spacer-5	AGUGGGGUCUGAGAGCUGCU
DR-PC-DNA1	AUCUGAGAAGUGGCAC**/PC Linker/dAdAdAdAdTdTdTdT**
DR-PC-DNA2	AUCUGAGAAGUGGCAC**/PC Linker/dGdCdGdCdGdCdGdC**
EBV PCR-F	ATGTCGTATTACACCATTGAGTCGT
EBV PCR-R	GCGTCTCCTAACAAGTTACATCAC
EBV PCR-R-FAM	**/56-FAM/**GCGTCTCCTAACAAGTTACATCAC
EBV RPA-F	GACCCGGCCCACAACCTGGCCCACTAAGGG
EBV RPA-R	ACTCCATCGTCAAAGCTGCACACAGTCACC
HCV RPA-F	GGG TCC TTT CTT GGA TAA ACC CGC TCA ATG C
HCV RPA-R	CTC GCA AGC ACC CTA TCA GGC AGT ACC ACA A
MPXV RPA-F	CTA ATG CGG AAT GTC AAC CTC TTC AA
MPXV RPA-R	AGA AAA TGT AGA TCC GGA AAT TAA TC
F-Q	**/56-FAM/**CCCCCCCC**/BHQ1/**
FAM-Biotin	**/56-FAM/**CCCCCCCC**/Biotin/**
FAM-ssDNA	**/56-FAM/**ACTGTTCATCGGCTG
DNA1	AAAATTTT
DNA2	CGCGCGCG

Abbreviations and modifications:

/PC Linker/: It links two nucleotide sequences via a short, UV-photocleavable C3 Spacer arm that can be attached at any position in the sequence.

/56-FAM/: 5' 6-FAM (Fluorescein) modification

/BHQ1/: Black Hole Quencher-1, which is used to quench green dye, such as FAM.

Supplementary Table 2. DNA target used for simulated detection in this work.

Name	Sequence (from 5’ to 3’)
EBV target	ATGTCGTATTACACCATTGAGTCGTCTCCCCTTTGGAATGGCCCCTGGACCCGGCCCACAACCTGGCCCACTAAGGGAGTCCATTGTCTGTTATTTCATGGTCTTTTTA**CAAACTCATATATTTGCTGA**GGTTTTGAAGGATGCGATTAAGGACCTTGTTATGACAAAGCCCGCTCCTACCTGCAATATCAAGGTGACTGTGTGCAGCTTTGACGATGGAGTAGATTTGCCTCCCTGGTTTCCACCTATGGTGGAAGGGGCTGCCGCGGAGGGTGATGACGGAGATGACGGAGATGAAGGAGGTGATGGAGATGAGGGTGAGGAAGGGCAGGAGTGATGTAACTTGTTAGGAGACGC
HCV target	GGGTCCTTTCTTGGATAAACCCGCTCAATGCCTGGAGATTTG**GGCGTGCCCCCGCAAGACTG**CTAGCCGAGTAGTGTTGGGTCGCGAAAGGCCTTGTGGTACTGCCTGATAGGGTGCTTGCGAG
MPXV target	CTAATGCGGAATGTCAACCTCTTCAATTAGAACACGGATCGTGTCAACCAGTTAAAGAAAAATACTCATTTGGGGAATATATGACTATCAACTGTGATGTTGGATATGAGGTTATTGGTGTTTC**GTATATAAGTTGTACGGCTA**ATTCTTGGAATGTTATTCCATCATGTCAACAAAAATGTGATATACCGTCCCTATCTAATGGATTAATTTCCGGATCTACATTTTCT
SEPT9-5mC-1 Target	GGCTGTCCACTCAGTCGGAGGTGAGGAACGACCTCCCTATCCCGTTGCCGGGTCCAAGCGGGGCC**/5mC/**GAGAGCCGCCGGGGAGAGCCAAAGGGAGGGGACCGATGGATTTCCCAGAGTGAAACTGTGCGTCCTGGAGAGTTCCGAGGCAGCCTCGCGAGCCCTCGAGGAGGTCGCTGTCGCTTGG
SEPT9-5mC-2 Target	GGCTGTCCACTCAGTCGGAGGTGAGGAACGACCTCCCTATCCCGTTGCCGGGTCCAAGCGGGGCC**/5mC/**GAGAGC**/5mC/**GCCGGGGAGAGCCAAAGGGAGGGGACCGATGGATTTCCCAGAGTGAAACTGTGCGTCCTGGAGAGTTCCGAGGCAGCCTCGCGAGCCCTCGAGGAGGTCGCTGTCGCTTGG
SEPT9-5mC-3 Target	GGCTGTCCACTCAGTCGGAGGTGAGGAACGACCTCCCTATCCCGTTGCCGGGTCCAAGCGGGGCC**/5mC/**GAGAGC**/5mC/**GC**/5mC/**GGGGAGAGCCAAAGGGAGGGGACCGATGGATTTCCCAGAGTGAAACTGTGCGTCCTGGAGAGTTCCGAGGCAGCCTCGCGAGCCCTCGAGGAGGTCGCTGTCGCTTGG
SEPT9-5mC-4 Target	GGCTGTCCACTCAGTCGGAGGTGAGGAACGACCTCCCTATCCCGTTGCCGGGTCCAAG**/5mC/**GGGGCC**/5mC/**GAGAGC**/5mC/**GC**/5mC/**GGGGAGAGCCAAAGGGAGGGGACCGATGGATTTCCCAGAGTGAAACTGTGCGTCCTGGAGAGTTCCGAGGCAGCCTCGCGAGCCCTCGAGGAGGTCGCTGTCGCTTGG
SEPT9-5mC-5 Target	GGCTGTCCACTCAGTCGGAGGTGAGGAACGACCTCCCTATCCCGTTGCCGGGTCCAAG**/5mC/**GGGG**/5mC/**C**/5mC/**GAGAGC**/5mC/**GC**/5mC/**GGGGAGAGCCAAAGGGAGGGGACCGATGGATTTCCCAGAGTGAAACTGTGCGTCCTGGAGAGTTCCGAGGCAGCCTCGCGAGCCCTCGAGGAGGTCGCTGTCGCTTGG
SEPT9-C-Target	GGCTGTCCACTCAGTCGGAGGTGAGGAACGACCTCCCTATCCCGTTGCCGGGTCCAAGCGGGGCCCGAGAGCCGCCGGGGAGAGCCAAAGGGAGGGGACCGATGGATTTCCCAGAGTGAAACTGTGCGTCCTGGAGAGTTCCGAGGCAGCCTCGCGAGCCCTCGAGGAGGTCGCTGTCGCTTGG

Abbreviations and modifications:

/5mC/: 5-methyl-2'-deoxycytidine

Supplementary Table 3. DNA and RNA sequences used for miRNA detection in this work.

Name	Sequence (from 5' to 3')
miR-17	CAAAGUGCUUACAGUGCAGGUAG
miR-21	UAGCUUAUCAGACUGAUGUUGA
miR-21 precursor	UGUCGGGUAGCUUAUCAGACUGAUGUUGACUGUUGAAUCUCAUGGCAACAC CAGUCGAUGGGCUGUCUGACA
miR-31	AGGCAAGAUGCUGGCAUAGCU
miR-92a	UAUUGCACUUGUCCCGGCCUGU
miR-429	UAAUACUGUCUGGUAAAACCGU
miR-4429	GCGGAGAGUCGGGUCGAAAA
F-21	GCCGCTAGCTTATCAGACTGA
Primer-21	GTCGTATCCAGTGCAGGGTCCGAGGTATTCGCACTGGATACGACTCAACA
R-primer	ATCCAGTGCAGGGTCCGAGG
F-17	CCTCTGCCAAAGTGCTTACAGTG
Primer-17	GTCGTATCCAGTGCAGGGTCCGAGGTATTCGCACTGGATACGACCTACCT
F-31	ACACTACGAGGCAAGATGCTGG
Primer-31	GTCGTATCCAGTGCAGGGTCCGAGGTATTCGCACTGGATACGACAGCTATG
F-92a	CCACCATGTATTGCACTTGTCCC
Primer-92a	GTCGTATCCAGTGCAGGGTCCGAGGTATTCGCACTGGATACGACACAGGC
U6 primer-F	CTCGCTTCGGCAGCACA
U6 primer-R	AACGCTTCACGAATTTGCGT
miR21 TF	GTTGTAAAACGACGGCCAGTTTTGTAGCTTATCAGACTGATGTTGACGG
miR21 TR	CCGTCAACATCAGTCTGATAAGCTACAAAACTGGCCGTCGTTTTACAAC
miR17 TF	GTTGTAAAACGACGGCCAGTTTTGCAAAGTGCTTACAGTGCAGGTAGCGG
miR17 TR	CCGCTACCTGCACTGTAAGCACTTTGCAAAACTGGCCGTCGTTTTACAAC
miR31 TF	GTTGTAAAACGACGGCCAGTTTTGAGGCAAGATGCTGGCATAGCTCGG
miR31 TR	CCGAGCTATGCCAGCATCTTGCCTCAAAACTGGCCGTCGTTTTACAAC
miR92a TF	GTTGTAAAACGACGGCCAGTTTTGTATTGCACTTGTCCCGGCCTGTCGG
miR92a TR	CCGACAGGCCGGGACAAGTGCAATACAAAACTGGCCGTCGTTTTACAAC

Supplementary Table 4. DNA sequence used for the simulation of single-base mutation detection in this work.

Name	Sequence (from 5' to 3')
Target WT	GTTGTAAAACGACGGCCAGTTTTGCAAACTCATATATTTGCTGACGG
Target WT-R	CCGTCAGCAAATATATGAGTTTGCAAAACTGGCCGTCGTTTTACAAC
Target M1	GTTGTAAAACGACGGCCAGTTTTGGAAACTCATATATTTGCTGACGG
Target M1-R	CCGTCAGCAAATATATGAGTTTCCAAAACTGGCCGTCGTTTTACAAC
Target M2	GTTGTAAAACGACGGCCAGTTTTGCTAACTCATATATTTGCTGACGG
Target M2-R	CCGTCAGCAAATATATGAGTTAGCAAAACTGGCCGTCGTTTTACAAC
Target M3	GTTGTAAAACGACGGCCAGTTTTGCATACTCATATATTTGCTGACGG
Target M3-R	CCGTCAGCAAATATATGAGTATGCAAAACTGGCCGTCGTTTTACAAC
Target M4	GTTGTAAAACGACGGCCAGTTTTGCAATCTCATATATTTGCTGACGG
Target M4-R	CCGTCAGCAAATATATGAGATTGCAAAACTGGCCGTCGTTTTACAAC
Target M5	GTTGTAAAACGACGGCCAGTTTTGCAAAGTCATATATTTGCTGACGG
Target M5-R	CCGTCAGCAAATATATGACTTTGCAAAACTGGCCGTCGTTTTACAAC
Target M6	GTTGTAAAACGACGGCCAGTTTTGCAAACACATATATTTGCTGACGG
Target M6-R	CCGTCAGCAAATATATGTGTTTGCAAAACTGGCCGTCGTTTTACAAC
Target M7	GTTGTAAAACGACGGCCAGTTTTGCAAACTGATATATTTGCTGACGG
Target M7-R	CCGTCAGCAAATATATCAGTTTGCAAAACTGGCCGTCGTTTTACAAC
Target M8	GTTGTAAAACGACGGCCAGTTTTGCAAACTCTTATATTTGCTGACGG
Target M8-R	CCGTCAGCAAATATAAGAGTTTGCAAAACTGGCCGTCGTTTTACAAC
Target M9	GTTGTAAAACGACGGCCAGTTTTGCAAACTCAAATATTTGCTGACGG
Target M9-R	CCGTCAGCAAATATTTGAGTTTGCAAAACTGGCCGTCGTTTTACAAC
Target M10	GTTGTAAAACGACGGCCAGTTTTGCAAACTCATTTATTTGCTGACGG
Target M10-R	CCGTCAGCAAATAAATGAGTTTGCAAAACTGGCCGTCGTTTTACAAC
Target M11	GTTGTAAAACGACGGCCAGTTTTGCAAACTCATAAATTTGCTGACGG
Target M11-R	CCGTCAGCAAATTTATGAGTTTGCAAAACTGGCCGTCGTTTTACAAC
Target M12	GTTGTAAAACGACGGCCAGTTTTGCAAACTCATATTTTTGCTGACGG
Target M12-R	CCGTCAGCAAAAATATGAGTTTGCAAAACTGGCCGTCGTTTTACAAC
Target M13	GTTGTAAAACGACGGCCAGTTTTGCAAACTCATATAATTGCTGACGG
Target M13-R	CCGTCAGCAATTATATGAGTTTGCAAAACTGGCCGTCGTTTTACAAC
Target M14	GTTGTAAAACGACGGCCAGTTTTGCAAACTCATATATATGCTGACGG
Target M14-R	CCGTCAGCATATATATGAGTTTGCAAAACTGGCCGTCGTTTTACAAC
Target M15	GTTGTAAAACGACGGCCAGTTTTGCAAACTCATATATTAGCTGACGG
Target M15-R	CCGTCAGCTAATATATGAGTTTGCAAAACTGGCCGTCGTTTTACAAC
Target M16	GTTGTAAAACGACGGCCAGTTTTGCAAACTCATATATTTCCTGACGG
Target M16-R	CCGTCAGGAAATATATGAGTTTGCAAAACTGGCCGTCGTTTTACAAC
Target M17	GTTGTAAAACGACGGCCAGTTTTGCAAACTCATATATTTGGTGACGG
Target M17-R	CCGTCACCAAATATATGAGTTTGCAAAACTGGCCGTCGTTTTACAAC
Target M18	GTTGTAAAACGACGGCCAGTTTTGCAAACTCATATATTTGCAGACGG
Target M18-R	CCGTCTGCAAATATATGAGTTTGCAAAACTGGCCGTCGTTTTACAAC
Target M19	GTTGTAAAACGACGGCCAGTTTTGCAAACTCATATATTTGCTCACGG
Target M19-R	CCGTGAGCAAATATATGAGTTTGCAAAACTGGCCGTCGTTTTACAAC
Target M20	GTTGTAAAACGACGGCCAGTTTTGCAAACTCATATATTTGCTGTCGG
Target M20-R	CCGAGAGCAAATATATGAGTTTGCAAAACTGGCCGTCGTTTTACAAC

Extended Data

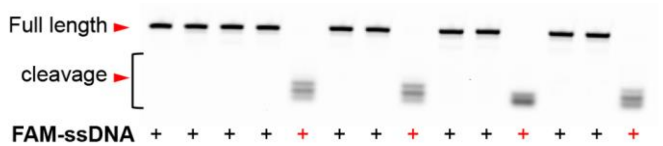

**FAM-ssDNA**	+	+	+	+	+	+	+	+	+	+	+	+	+	+
Cas12b	-	+	+	+	+	+	+	+	+	+	+	+	+	+
sgRNA	-	-	+	+	+	-	-	-	-	-	-	-	-	-
tracrRNA + crRNA	-	-	-	-	-	+	+	+	-	-	-	-	-	-
tracrDR + Spacer	-	-	-	-	-	-	-	-	+	+	+	-	-	-
tracrRNA+DR + Spacer	-	-	-	-	-	-	-	-	-	-	-	+	+	+
Non-target	-	-	-	+	-	-	+	-	-	+	-	-	+	-
ssDNA target	-	-	-	-	+	-	-	+	-	-	+	-	-	+
Lane	1	2	3	4	5	6	7	8	9	10	11	12	13	14

Supplementary Fig. 1. Denaturing PAGE of the *trans*-cleavage activity of four sgRNA types. Denaturing PAGE analysis confirms the effective *trans*-cleavage activity of tracrDR+Spacer and tracrRNA+DR+Spacer, comparable to that of full-length sgRNA and tracrRNA+crRNA. FAM-ssDNA denotes a FAM-labeled single-stranded random DNA strand that is cleaved upon activation of the *trans*-cleavage activity of the Cas12b system. ssDNA target refers to the single-stranded target DNA that is targeted by the sgRNA, whereas Non-target indicates single-stranded DNA that is not recognized by sgRNA.

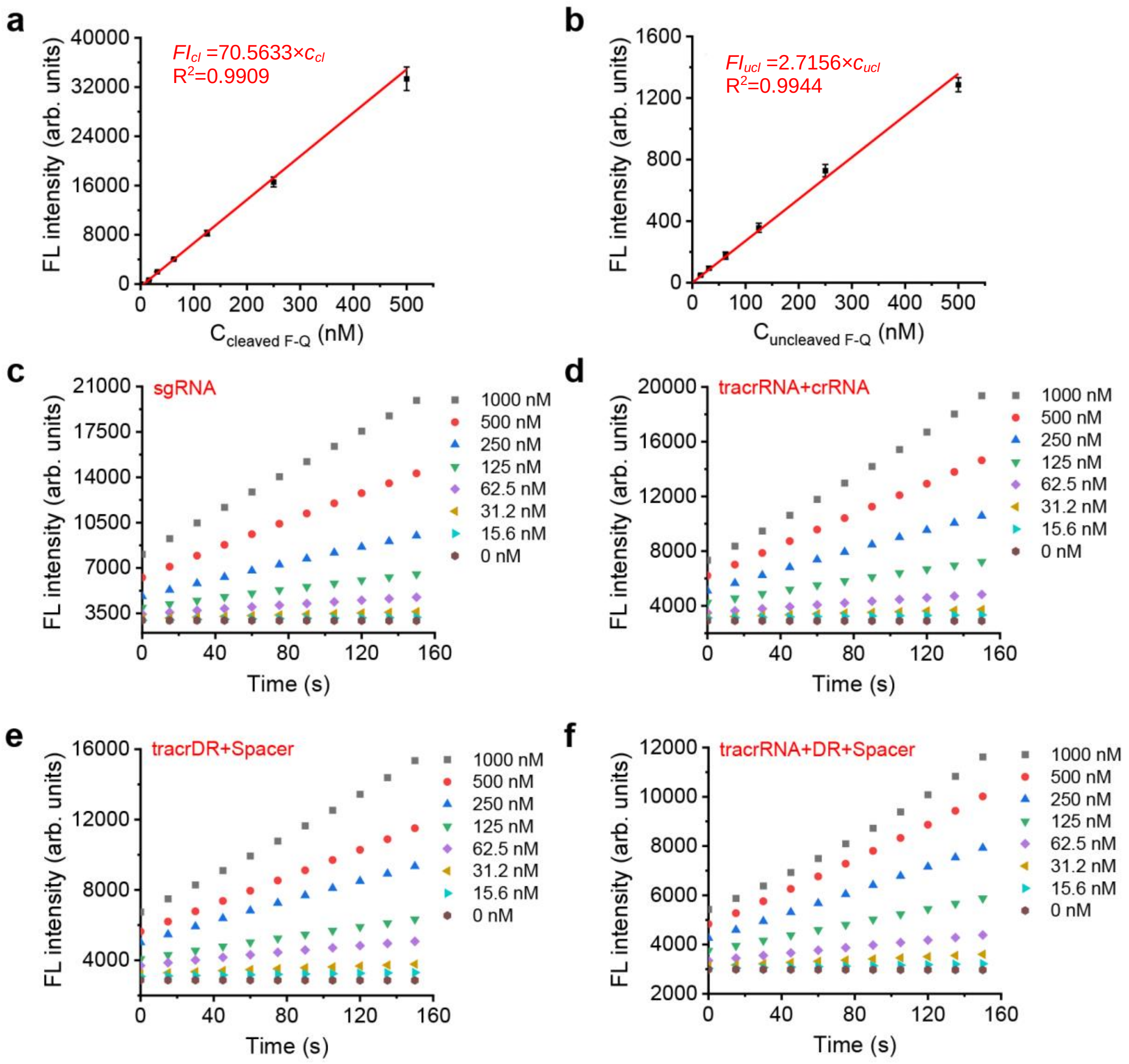

Supplementary Fig. 2. Kinetic analysis of four sgRNA types guided *trans*-cleavage. (a) Subtracted fluorescence (FI_{cl}) plotted against the concentration of cleaved F-Q (c_{cl}). Error bars indicate the mean value +/− SD (n = 3) of biologically independent samples. (b) Subtracted fluorescence (FI_{ucl}) plotted against the concentration of uncleaved F-Q (c_{ucl}). Error bars indicate the mean value +/− SD (n = 3) of biologically independent samples. (c-f) Subtracted fluorescence plotted against time for varying concentrations of F-Q, ranging from 16.5 nM to 1000 nM, with 25 nM Cas12b, 1 nM activators, 100 nM four types of sgRNA: (c) sgRNA; (d) tracrRNA+crRNA; (e) tracrDR+Spacer; (f) tracr+DR+Spacer, and incubated for continuous fluorescence detection for 150 s at 48 °C.

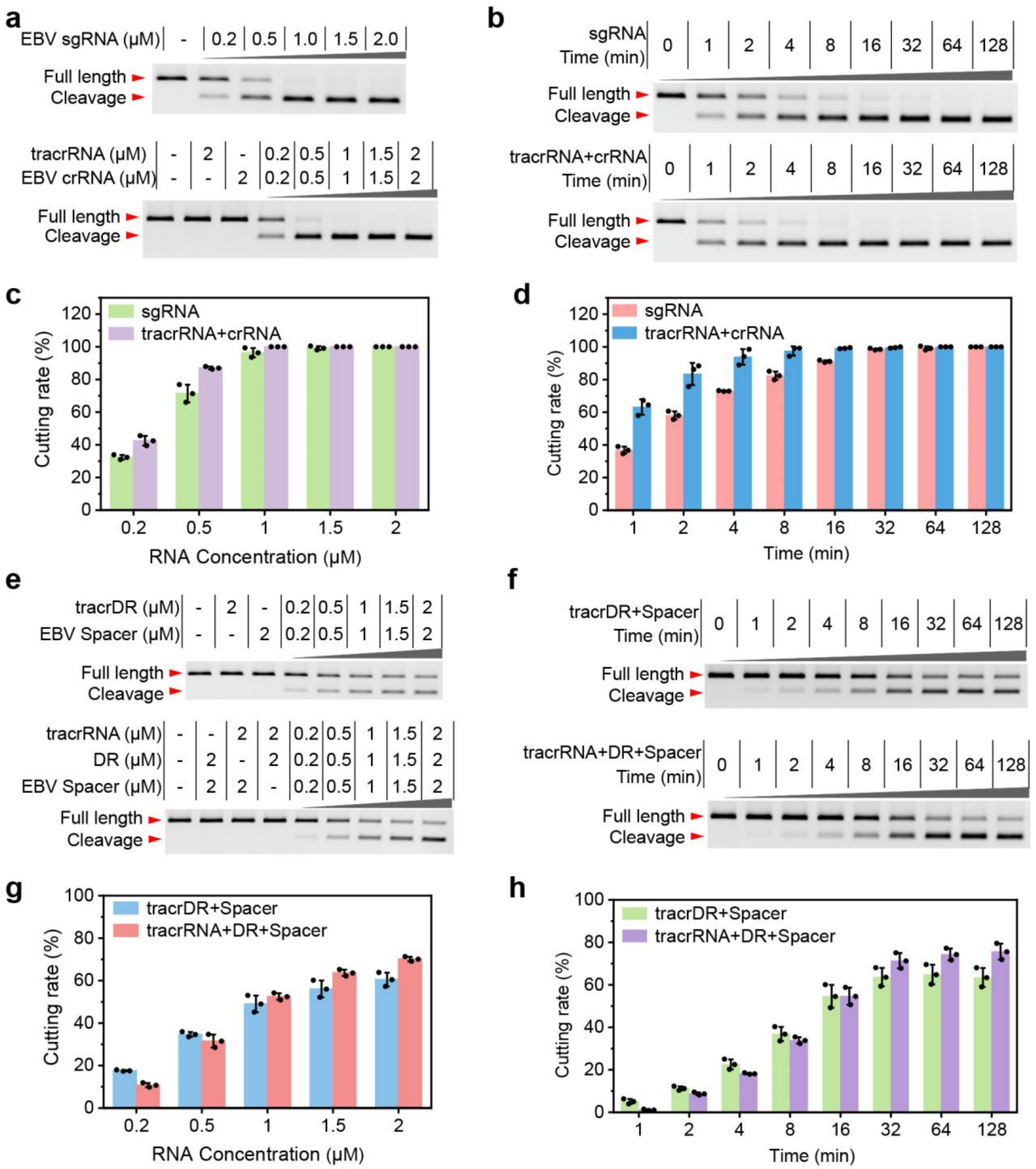

Supplementary Fig. 3. Comparative analysis of the *cis*-cleavage activity of four sgRNA types at different concentrations and reaction time. (a) Comparative analysis of the *cis*-cleavage activity of full-length sgRNA or tracrRNA+crRNA-assisted Cas12b system at different concentrations using 2% agarose gel electrophoresis. FAM-labeled EBV target (20 nM), Cas12b (200 nM), and varying concentrations of sgRNA or tracrRNA+crRNA (0-2 μM) were incubated at 48°C for 128 min. (b) Comparative analysis of the *cis*-cleavage activity of full-length sgRNA or tracrRNA+crRNA-assisted Cas12b at the same

concentration (2 μM) and different reaction times using 2% agarose gel electrophoresis. FAM-labeled EBV target (20 nM), Cas12b (200 nM), and full-length sgRNA or tracrRNA+crRNA (2 μM) were incubated at 48°C for 0-128 min. (c) Bar graph quantification of the *cis*-cleavage activity of Cas12b with increasing concentrations of full-length sgRNA or tracrRNA+DR+Spacer. (d) Bar graph quantification of the cleavage variation of Cas12b with the same concentration (2μM) of full-length sgRNA or tracrRNA+crRNA and prolonged reaction time. (e) Comparative analysis of Cas12b's *cis*-cleavage activity assisted by different concentrations of tracrDR+Spacer or tracrRNA+DR+Spacer using 2% agarose gel electrophoresis. FAM-labeled EBV target (20 nM), Cas12b (200 nM), and various concentrations of tracrDR+Spacer or tracrRNA+DR+Spacer were incubated at 48°C for 128 min. The cleavage efficiency increased gradually with higher concentrations of split sgRNA. (f) Comparative analysis of Cas12b's cleavage activity assisted by the same concentration (2 μM) of tracrDR+Spacer or tracrRNA+DR+Spacer at different reaction times using 2% agarose gel electrophoresis. FAM-labeled EBV target (20 nM), Cas12b (200 nM), and split sgRNA (2 μM) were incubated at 48°C for 0-128 min to validate the increase in cleavage with prolonged incubation time, reaching a plateau at 32 min. (g) Bar chart quantitatively analyzing the increase in Cas12b's *cis*-cleavage activity with the increasing concentration of tracrDR+Spacer or tracrRNA+DR+Spacer. (h) Bar chart quantitatively analyzing the change in Cas12b's cleavage with prolonged reaction time for the same concentration of tracrDR+Spacer or tracrRNA+DR+Spacer. All the experiments were conducted in triplicate and error bars represent mean value +/− SD (n = 3). Source data are provided as a Source Data file.

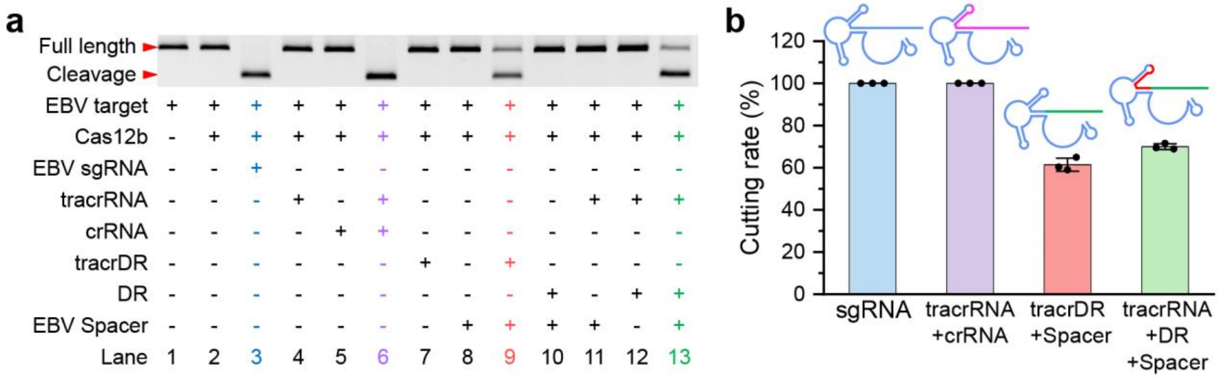

Supplementary Fig. 4. Analysis of Cas12b's *cis*-cleavage activity assisted by different sgRNA types. (a) 2% agarose gel electrophoresis tested sgRNA types include EBV sgRNA, tracrRNA+EBV crRNA, tracrDR+EBV Spacer, and tracrRNA+DR+EBV Spacer. These were incubated with FAM-labeled EBV target (20 nM) and Cas12b (200 nM) at 48°C for 128 min for validation. (b) Bar chart quantitatively comparing the cleavage activity of different sgRNA types relative to full-length sgRNA. All the experiments were conducted in triplicate and error bars represent mean value +/− SD (n = 3).

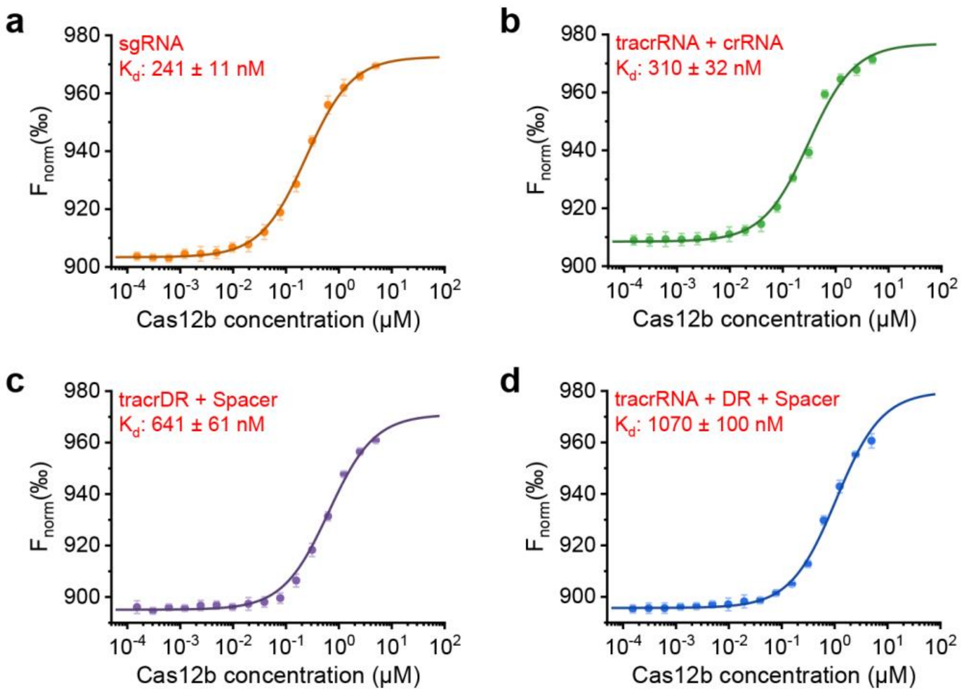

Supplementary Fig. 5. Microscale thermophoresis assays comparing the interaction between the Cas12b–sgRNA complex. Binding curves illustrating the response values as a function of Cas12b concentration, determined using the Monolith Pico (NanoTemper) system, for the measurement of dissociation constants between Cas12b and (a) sgRNA, (b) tracrRNA+crRNA, (c) tracrDR+Spacer, and (d) tracrRNA+DR+Spacer. All the experiments were conducted in triplicate and error bars represent mean value +/− SD (n = 3). Source data are provided as a Source Data file.

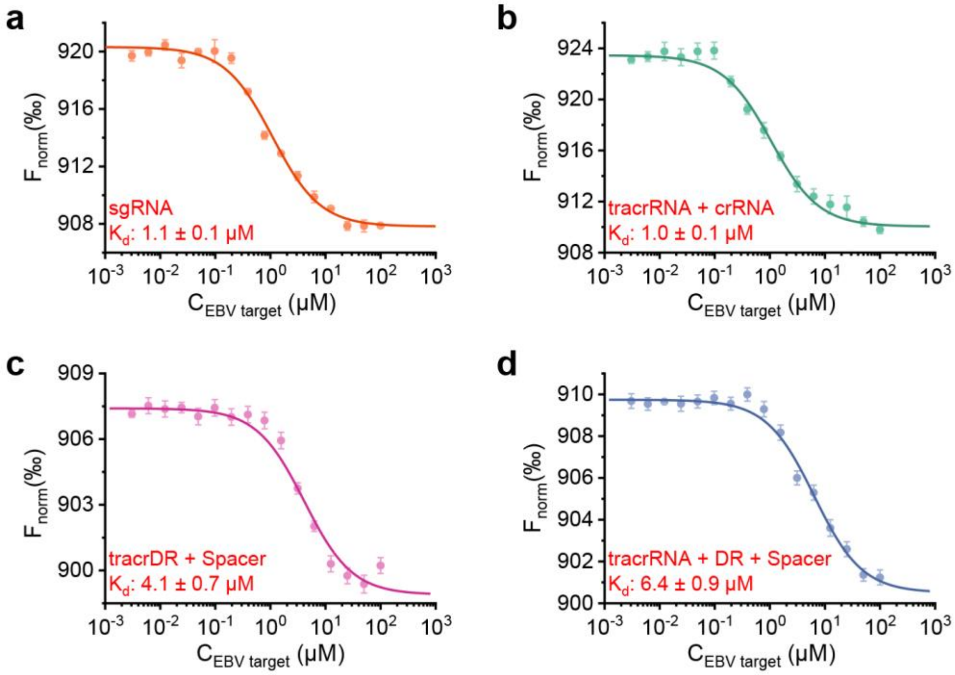

Supplementary Fig. 6. Microscale thermophoresis assays comparing the interaction between the Cas12b–sgRNA–Targets complex. Binding curves depicting the response values in relation to the concentration of target nucleic acid for the either split or full-length sgRNA-Cas12b complexes, determined using the Monolith Pico (NanoTemper) system, to measure the dissociation constants of either split or full-length sgRNA-Cas12b complexes with target DNA. The curves are shown for (a) sgRNA, (b) tracrRNA+crRNA, (c) tracrDR+Spacer, and (d) tracrRNA+DR+Spacer. Error bars indicate the standard deviation (n = 3). All the experiments were conducted in triplicate and error bars represent mean value +/− SD (n = 3). Source data are provided as a Source Data file.

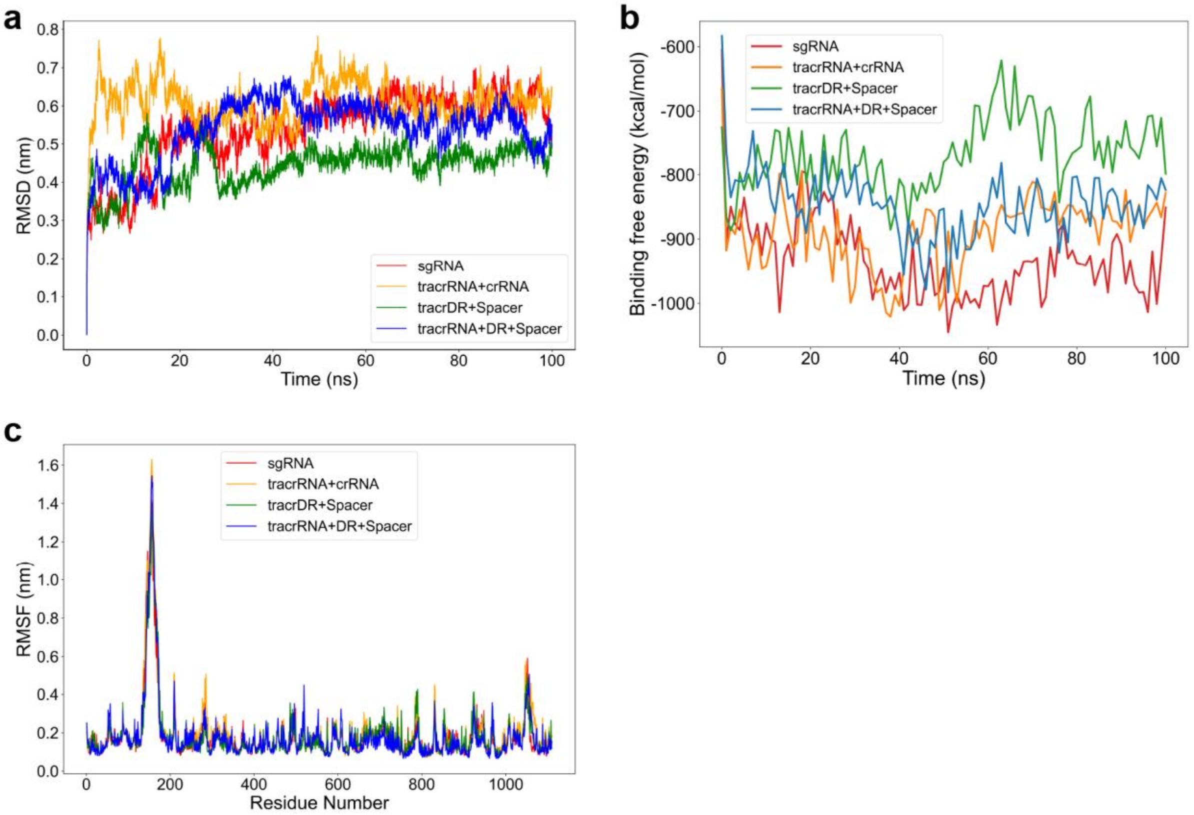

Supplementary Fig. 7. Molecular dynamics simulation analysis of Cas12b interactions with full length sgRNA or split sgRNA. (a) Time-dependent root-mean-square deviation (RMSD) of sgRNA, tracrRNA+crRNA, tracrDR+Spacer, and tracrRNA+DR+Spacer bound to Cas12b during the 0–100 ns simulation. All systems exhibit stable RMSD values after 30 ns, indicating reliable and meaningful simulations within the 100 ns timeframe. (b) Time-dependent binding free energy of sgRNA, tracrRNA+crRNA, tracrDR+Spacer, and tracrRNA+DR+Spacer bound to Cas12b during the 0–100 ns simulation. Full-length sgRNA demonstrates the lowest binding free energy, suggesting the strongest interaction with Cas12b. (c) Root-mean-square fluctuation (RMSF) analysis of Cas12b amino acid residues upon binding to full-length or split sgRNA. The y-axis represents RMSF (in nm), and the x-axis denotes the residue number. Higher RMSF values indicate greater conformational flexibility or reduced stability. Notably, residues 100–200 across all four systems exhibit elevated RMSF, suggesting increased flexibility or lower stability in this region. Source data are provided as a Source Data file.

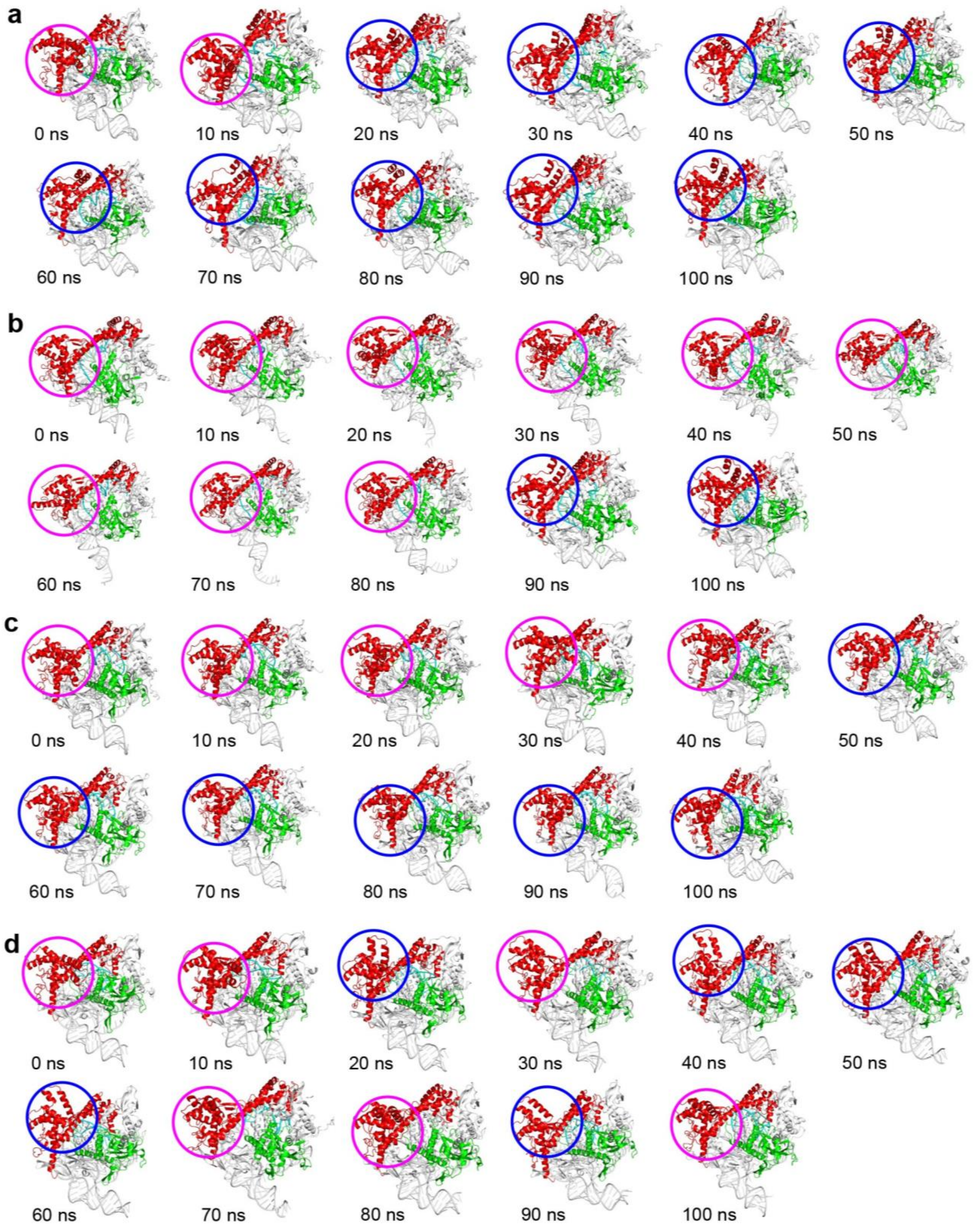

Supplementary Fig. 8. Conformational snapshots of Cas12b interactions with full length sgRNA or split sgRNA during molecular dynamics simulations (0–100 ns, sampled every 10 ns). (a) sgRNA, (b) tracrRNA+crRNA, (c) tracrDR+Spacer, and (d) tracrRNA+DR+Spacer. Key structural features are

highlighted: the REC1 domain of Cas12b is shown in red, the RuvC domain in green, and the Spacer region of sgRNA in cyan. Conformations before structural stabilization are marked with magenta circle, while blue circles indicate more stable conformations observed after stabilization. This visualization provides insights into the dynamic behavior and structural transitions of the Cas12b-sgRNA complexes during the simulation.

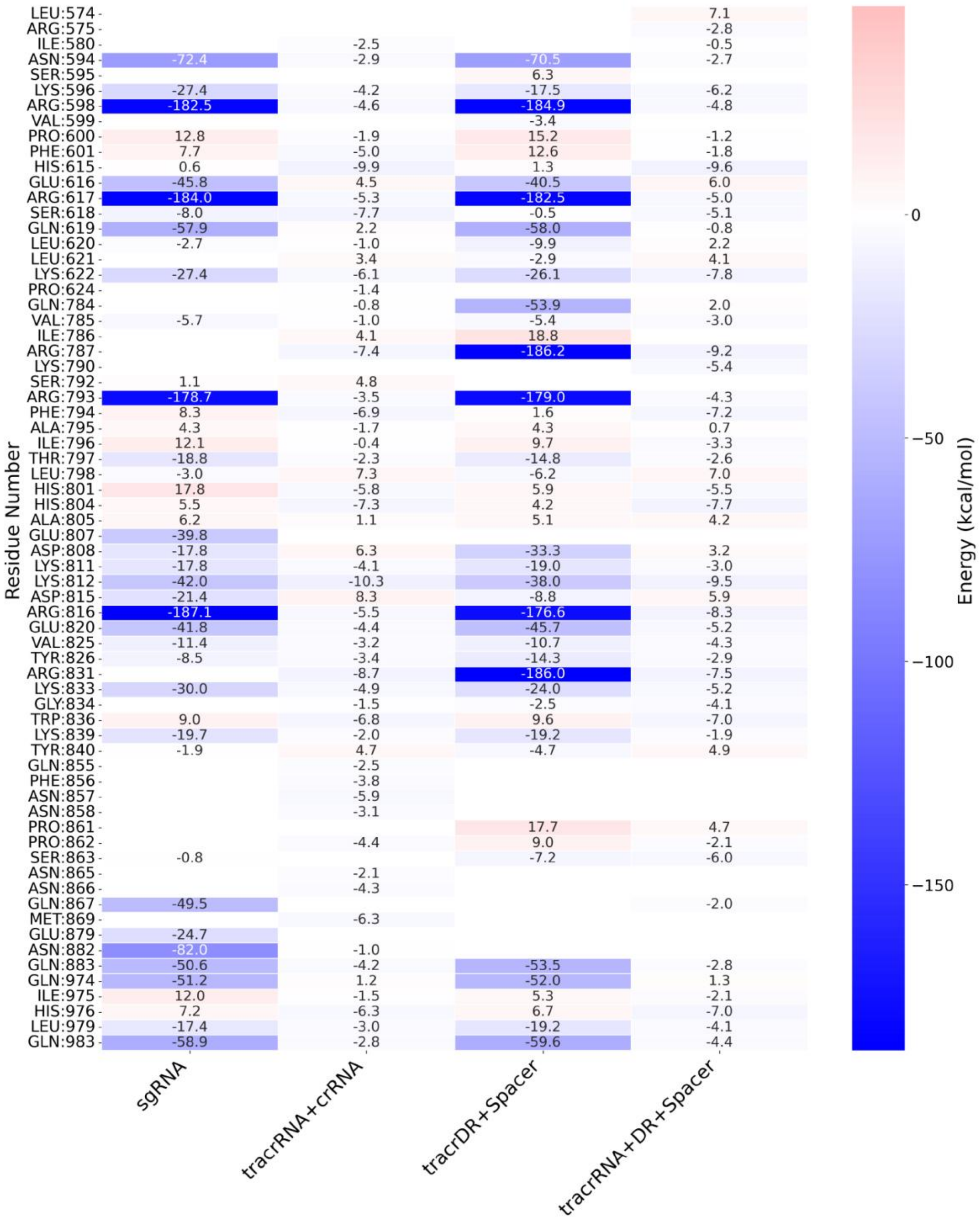

Supplementary Fig. 9. Energy contribution profiles of Spacer regions to Cas12b RuvC domain residues in molecular dynamics simulations. The analysis includes sgRNA, tracrRNA+crRNA, tracrDR+Spacer, and tracrRNA+DR+Spacer. Notably, the split sgRNA exhibits significant variations in energy contributions to RuvC domain residues compared to full-length configurations, highlighting distinct interaction patterns.

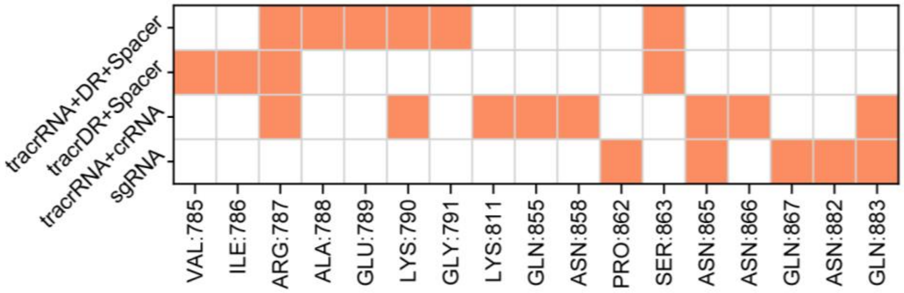

Supplementary Fig. 10. Distribution of amino acid residues involved in hydrogen bonding and electrostatic interactions between the RuvC domain and Spacer region in full-length and split sgRNA complexes with Cas12b.

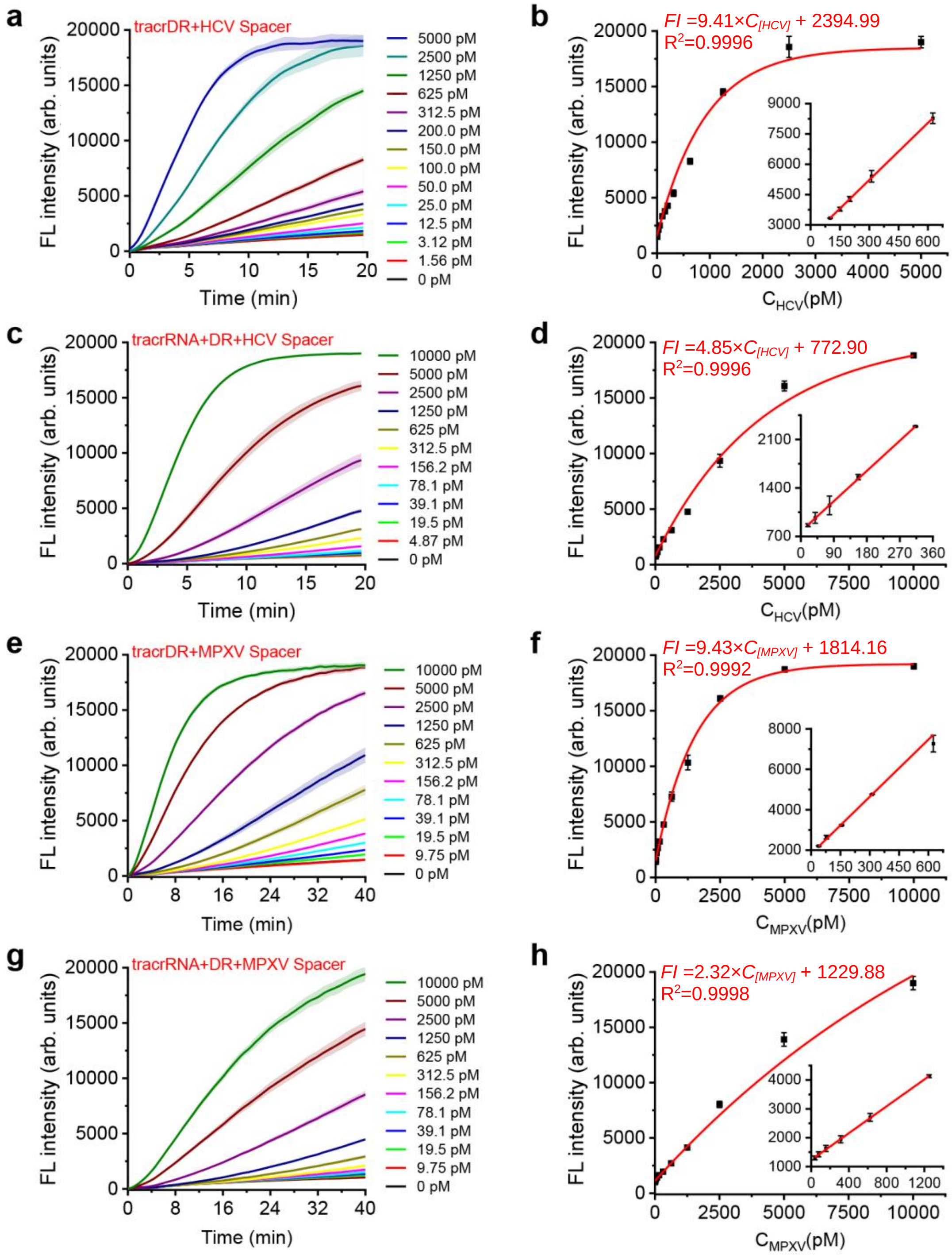

Supplementary Fig. 11. Quantitative detection of various nucleic acid targets using a universal split sgRNA strategy with matching spacers. The tracrDR+Spacer strategy was tested with HCV-specific Spacers against various concentrations of HCV targets, yielding fluorescence detection values (a) and their corresponding linear relationship plot (b). Similarly, the tracrRNA+DR+Spacer strategy was assessed using the HCV Spacer with varying concentrations of HCV targets, as indicated by the fluorescence detection values (c)

and the corresponding linear relationship plot (d). The tracrDR+Spacer strategy was tested with MPXV-specific Spacers against various concentrations of MPXV targets, yielding fluorescence detection values (e) and their corresponding linear relationship plot (f). Similarly, the tracrRNA+DR+Spacer strategy was assessed using the MPXV Spacer with varying concentrations of MPXV targets, as indicated by the fluorescence detection values (g) and the corresponding linear relationship plot (h). All the experiments were conducted in triplicate and error bars represent mean value +/− SD (n = 3). Source data are provided as a Source Data file.

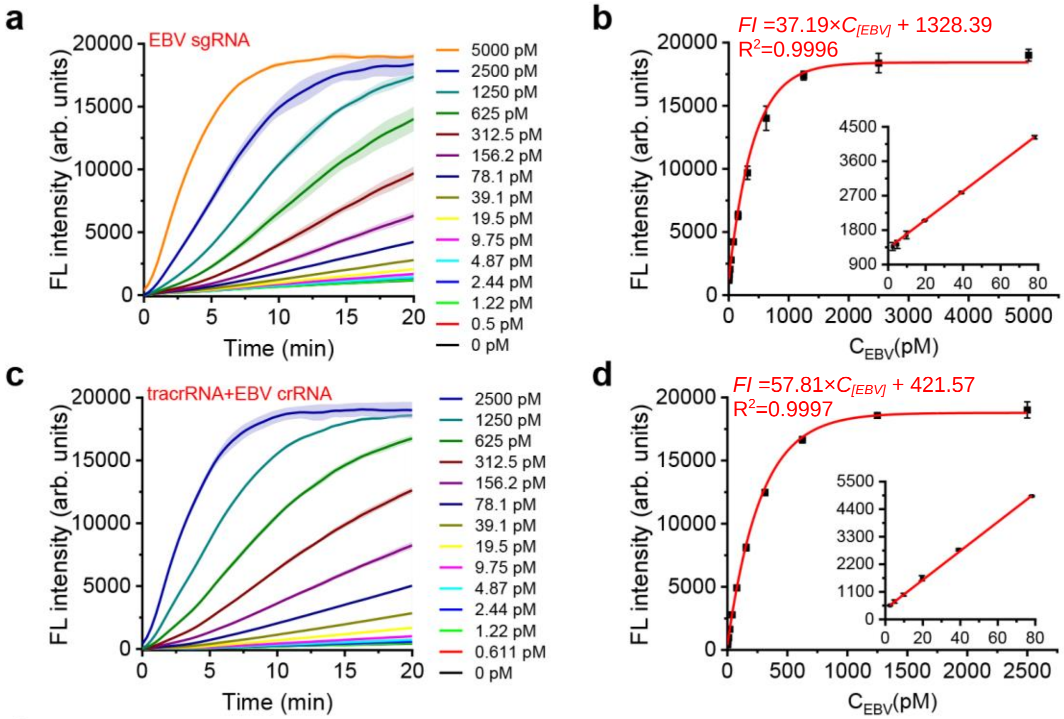

e

	Cas12b	*Linear range*	*R^2*	*LOD*
EBV	sgRNA	2.5-80 pM	0.9996	0.556 pM
	tracrRNA+crRNA	2.5-80 pM	0.9997	0.830 pM
	tracrDR+Spacer	12.5-200 pM	0.9983	3.18 pM
	tracrRNA+DR+Spacer	40-625 pM	0.9960	15.9 pM

Supplementary Fig. 12. Quantitative detection of EBV targets using four sgRNA types. The full-length sgRNA strategy designed to target EBV against varying concentrations of EBV targets, as indicated by the fluorescence detection values (a) and the corresponding linear relationship plot (b). Similarly, the tracrRNA+EBV crRNA strategy designed for EBV target against various concentrations of EBV targets, as indicated by the fluorescence detection values (c) and the corresponding linear relationship plot (d). (e) A summary table showing the linear ranges and detection limits for EBV targets as determined by the full-

length sgRNA, tracrRNA+crRNA tracrDR+Spacer, and tracrRNA+DR+Spacer strategies. All the experiments were conducted in triplicate and error bars represent mean value +/− SD (n = 3). Source data are provided as a Source Data file.

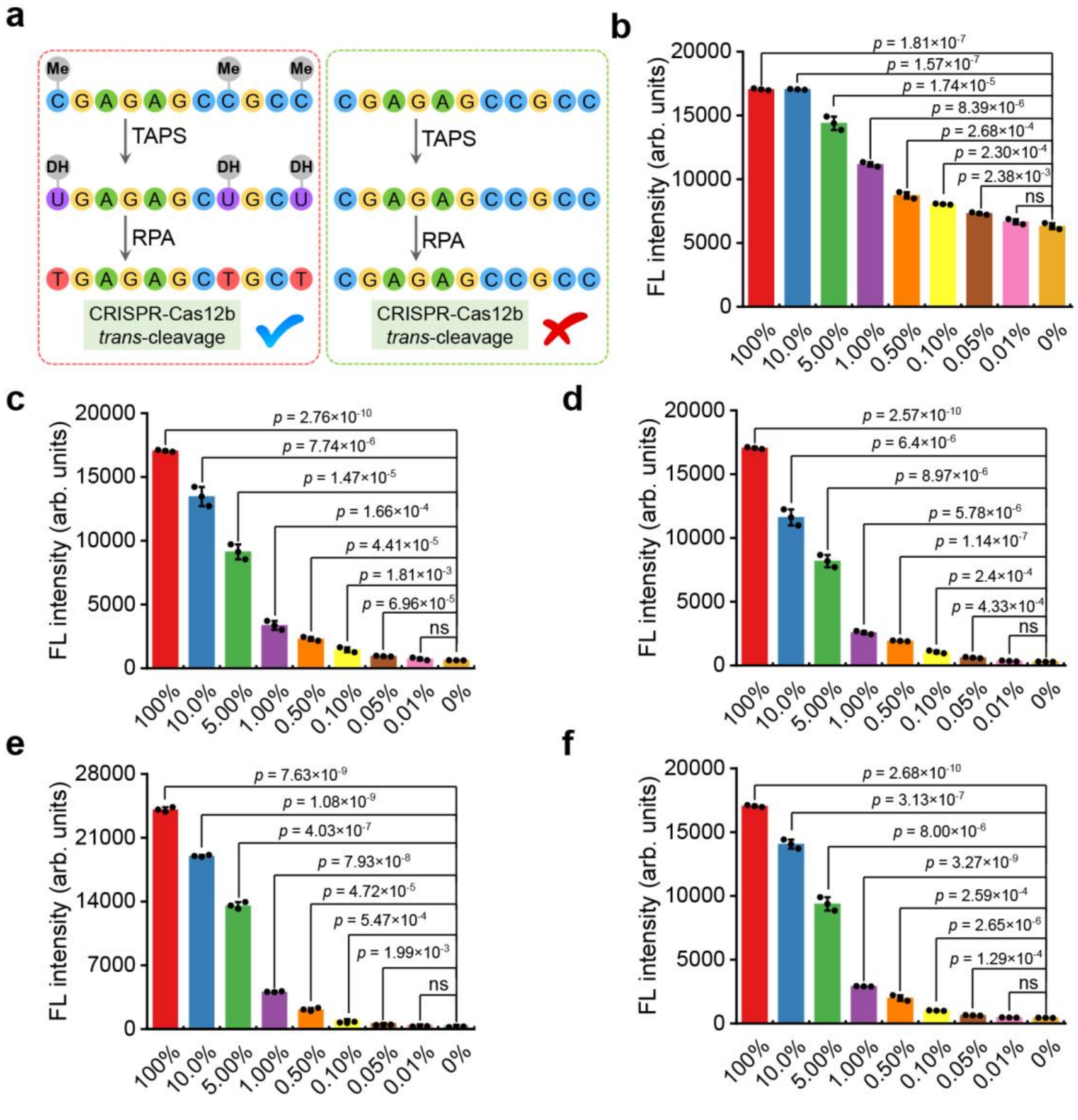

Supplementary Fig. 13. Detection of colorectal cancer methylation of Septin9 using the split sgRNA-assisted CRISPR-Cas12b system. (a) Schematic representation of the tracrRNA+DR+Spacer-assisted CRISPR-Cas12b system for the detection of methylation sites. Methylated Septin9 undergoes selective oxidation of 5mC to generate DHU under the action of TAPS method, while unmethylated C remains unchanged.[1] Subsequently, under the action of RPA, the originally methylated sites are selectively converted to T. Unmethylated Septin9 remains unchanged under the TAPS method. We can then detect it using the tracrRNA+DR+SEPT9 Spacer assisted CRISPR-Cas12b system. (b-f) Bar graph analysis of the

detection of different ratios of methylated/unmethylated Septin9 using this method. (b) Target nucleic acid with one methylated site, (c) two, (d) three, (e) four, and (f) five methylated sites. Our research demonstrates that split sgRNA assisted Cas12b, with its exceptional single-base resolution in detecting target nucleic acids, can accurately identify differences even when the target sequence contains only a single methylation site. All the experiments were conducted in triplicate and error bars represent mean value +/− SD (n = 3), and statistical analysis was conducted using a two-tailed t-test. Source data are provided as a Source Data file. This Supplementary Fig. 13a is adapted from Wang, J., Zhang, W., Li, W., Xie, Q., Zang, Z. and Liu, C. Enhancement of CRISPR-Cas12a system through universal circular RNA design. *Cell Rep. Methods* 5, 101076 (2025),[2] licensed under CC-BY 4.0 (https://creativecommons.org/licenses/by/4.0/).

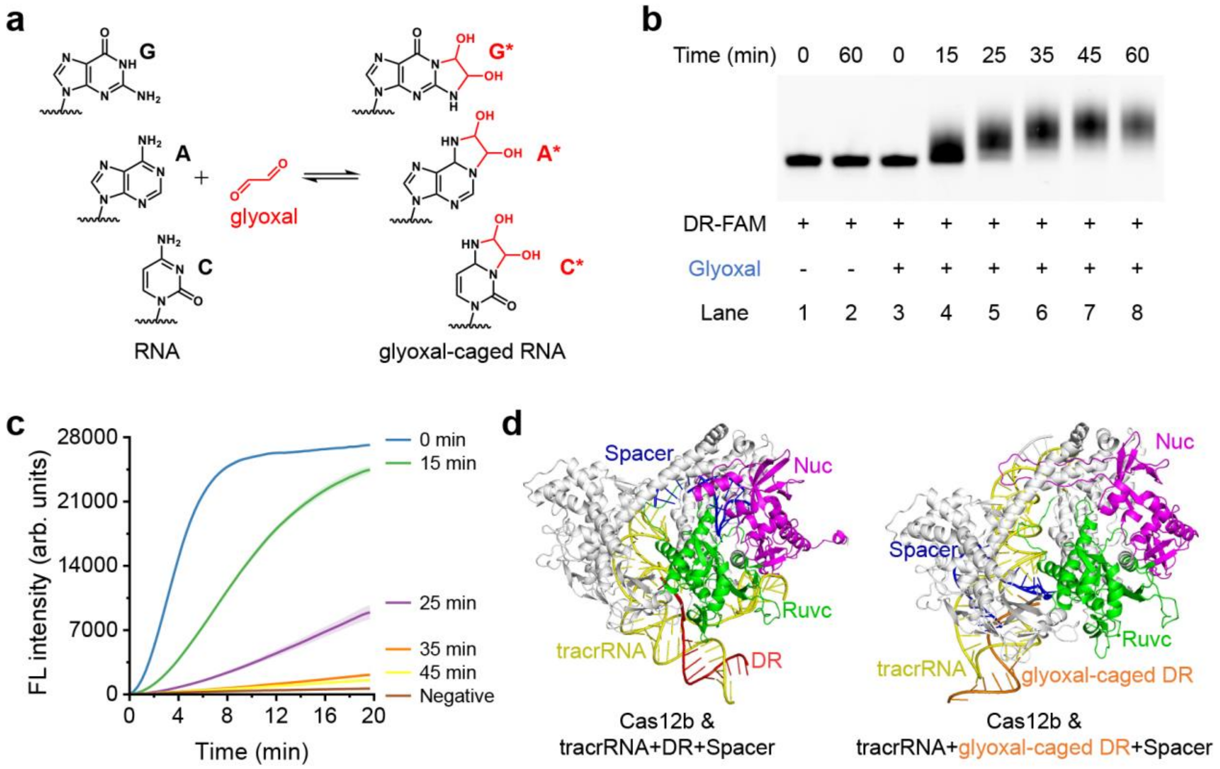

Supplementary Fig. 14. Structural and functional characterization of DR-glyoxal adduct formation and its impact on Cas12b binding. (a) Schematic illustration of the molecular structure of DR-glyoxal, formed through the reaction of RNA bases on DR with glyoxal. (b) Gel electrophoresis analysis of FAM-labeled DR following its reaction with glyoxal over a time course of 0–60 minutes. The results demonstrate successful formation of the DR-glyoxal adduct after 45 minutes of incubation. (c) Real-time fluorescence graphs showing the inhibition of *trans*-cleavage activity in the tracrRNA+DR+Spacer assisted CRISPR-

Cas12b system after different durations of glyoxal incubation with DR region. Data represent the mean and standard deviation of three technical replicates. Data are shown as mean value +/− SD (n = 3, biologically independent samples). (d) Molecular dynamics simulations depicting the conformational interactions between Cas12b and the tracrRNA+DR+Spacer complex, compared with the tracrRNA+DR-glyoxal adduct+Spacer complex. Key structural features are highlighted, revealing that the DR-glyoxal adduct disrupts the normal binding of the tracrRNA+DR+Spacer complex to Cas12b, leading to altered conformational dynamics. Source data are provided as a Source Data file.

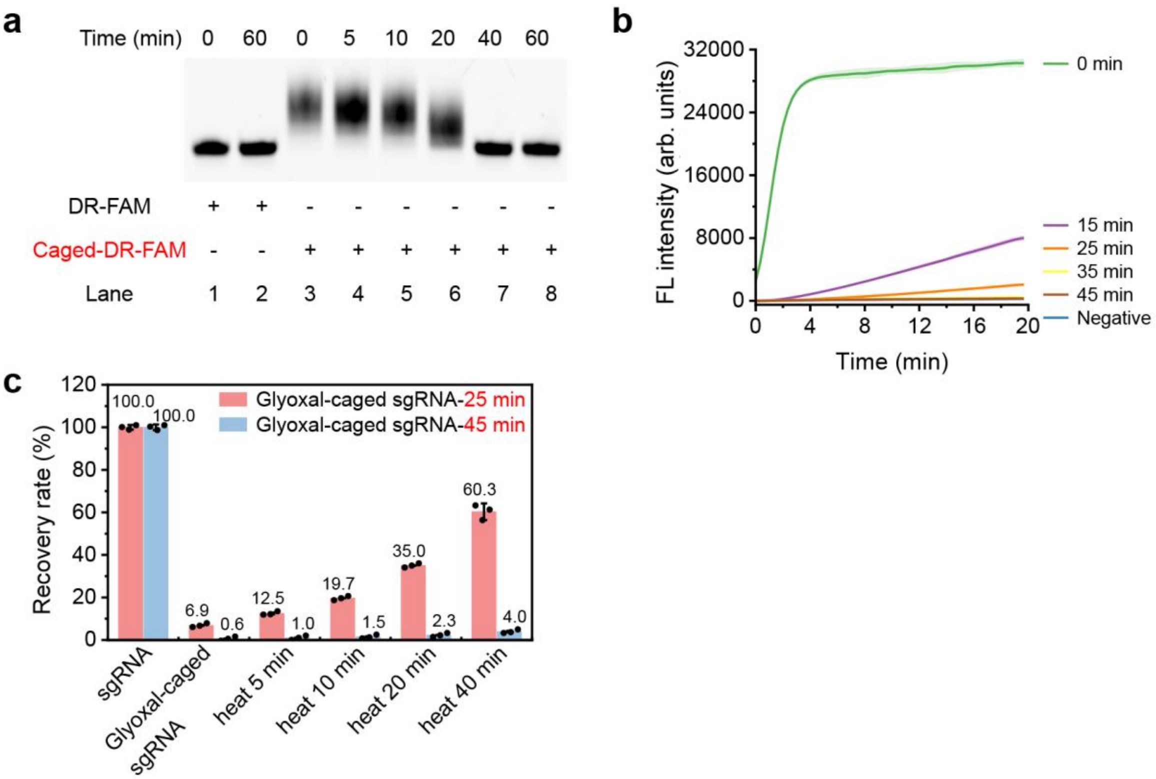

Supplementary Fig. 15. Thermal recovery of RNA-glyoxal adduct and its impact on CRISPR-Cas12b trans-cleavage activity. (a) Gel electrophoresis analysis of the DR-glyoxal adduct heated at 60°C over a time course of 0–60 minutes, demonstrating the complete recovery of the DR structure after 40 minutes of heating. (b) Real-time fluorescence graphs showing the inhibition of *trans*-cleavage activity in the full-length sgRNA assisted CRISPR-Cas12b system after different durations of glyoxal incubation with full-length sgRNA. Data represent the mean and standard deviation of three technical replicates. (c) Bar graph quantification of the recovery rate (%) of Cas12b's *trans*-cleavage activity at 60°C under 25-minute and 45-minute glyoxal caged sgRNA, relative to the heating time at 60°C. The 45-minute glyoxal caged sgRNA exhibits only 3.98% recovery after heating at 60°C for 40 min, while the 45-minute glyoxal caged DR achieves 96.5% recovery. Glyoxal caged longer RNA such as full-length sgRNA shows even poorer

recovery rate in restoring the *trans*-cleavage activity of the CRISPR-Cas12b system after heating. All the experiments were conducted in triplicate and error bars represent mean value +/− SD (n = 3). Source data are provided as a Source Data file.

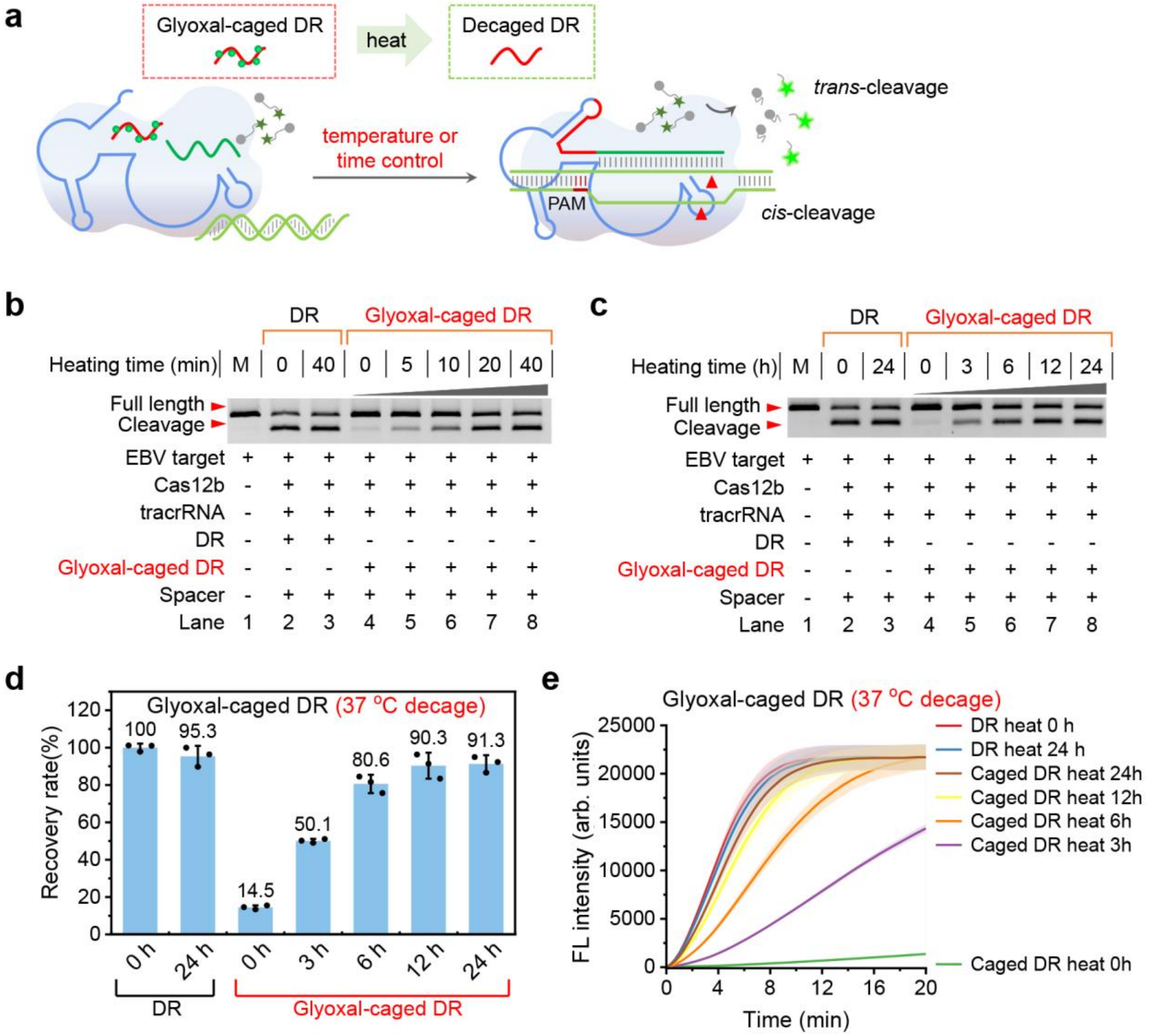

Supplementary Fig. 16. Controlled cleavage activity of CRISPR-Cas12b system using glyoxal-caged RNA. (a) Illustration of glyoxal caged DR's effective inhibition of CRISPR-Cas12b's *cis*- and *trans*-cleavage activity in the tracrRNA+DR+Spacer strategy. The function can be precisely restored by temperature or time-based regulation. (b) Gel electrophoresis validation of the inhibition and recovery of CRISPR-Cas12b's *cis*-cleavage activity by glyoxal caged DR through temperature control. Glyoxal caged DR effectively inhibits CRISPR-Cas12b's *cis*-cleavage activity (0 min), gradually regaining activity after incubation at 60°C for 5 min, and reaching nearly complete recovery at 40 min. (c) Gel electrophoresis validation of the inhibition and recovery of CRISPR-Cas12b's *cis*-cleavage activity by glyoxal caged DR through incubation at physiological temperature for different durations. Glyoxal caged DR effectively

inhibits CRISPR-Cas12b's *cis*-cleavage activity (0 h), with gradual recovery observed after incubation at physiological temperature for 3 hours, and almost complete recovery at 12-24 hours. (d) Bar graph showing the relationship between recovery rate of *cis*-cleavage activity and incubation time for glyoxal-caged DR at physiological temperature. (e) Inhibition of CRISPR-Cas12b's *trans*-cleavage activity by glyoxal caged DR, with precise recovery achieved by incubation at physiological temperature (37°C) for a specific duration. Glyoxal treated DR inhibits CRISPR-Cas12b's *trans*-cleavage activity (0 h), with gradual recovery observed after incubation at physiological temperature for 3 hours, and almost complete recovery at 12-24 hours. All the experiments were conducted in triplicate and error bars represent mean value +/− SD (n = 3). Source data are provided as a Source Data file. This Supplementary Fig. 16a is adapted from Wang, J., Zhang, W., Li, W., Xie, Q., Zang, Z. and Liu, C. Enhancement of CRISPR-Cas12a system through universal circular RNA design. *Cell Rep. Methods* 5, 101076 (2025),[2] licensed under CC-BY 4.0 (https://creativecommons.org/licenses/by/4.0/).

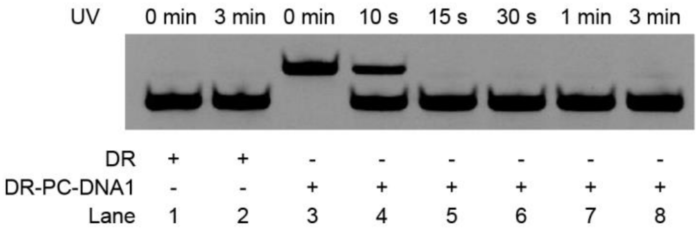

Supplementary Fig. 17. Denaturing PAGE validation of the UV (365 nm, 35 W) irradiation time of DR-PC-DNA1 and PC linker breakage.

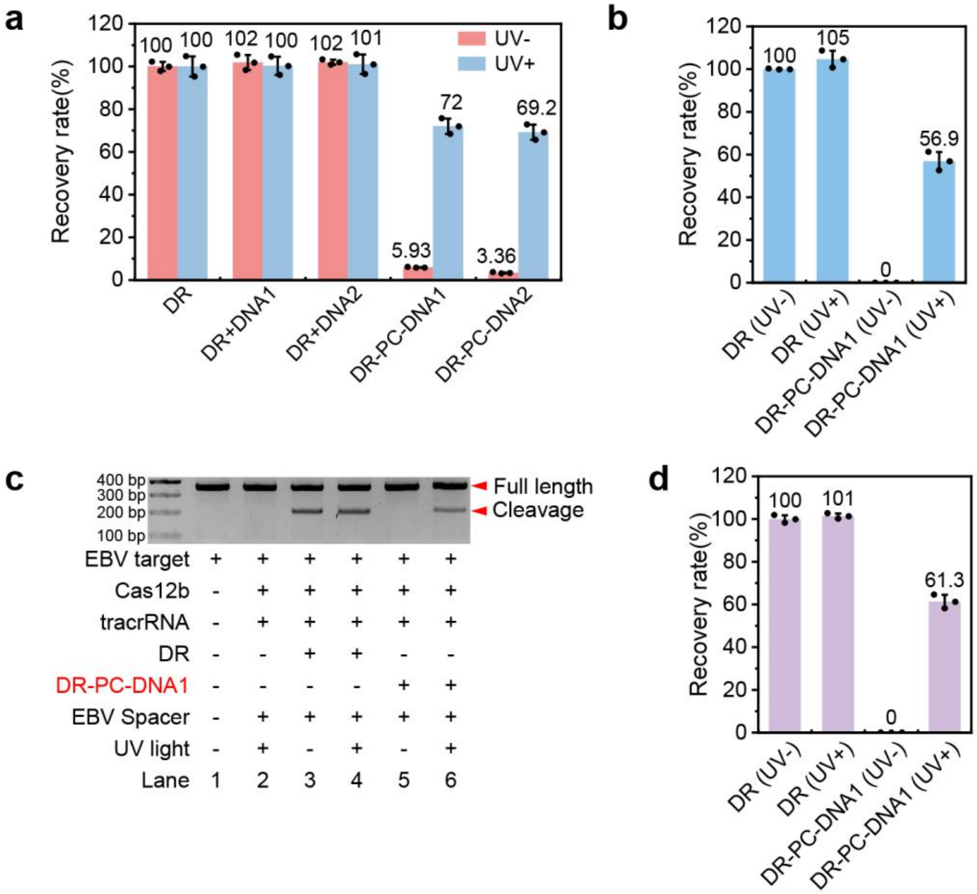

Supplementary Fig. 18. Photo-controlled *cis*-cleavage activity of CRISPR-Cas12b system using DR-PC-DNA. (a) Bar graph quantification of the effective inhibition of CRISPR-Cas12b's *trans*-cleavage activity by DR-PC-DNA1 or DR-PC-DNA2, and with activity recovery after 15 seconds of UV light exposure, reaching 72% (DR-PC-DNA1) and 69.2% (DR-PC-DNA2) recovery rates. (b) Bar graph quantification of the effective inhibition of CRISPR-Cas12b's *cis*-cleavage activity by DR-PC-DNA1 at 37°C, and with 56.9% activity recovery after 15 seconds of UV light (365 nm, 35 W) exposure. Moreover, the 15-second UV light exposure has no impact on the system. (c) Gel electrophoresis confirming the effective inhibition of CRISPR-Cas12b's *cis*-cleavage activity at 48°C by DR-PC-DNA1, and with activity recovery after 15 seconds of UV light exposure. (d) Bar graph quantification of the effective inhibition of CRISPR-Cas12b's *cis*-cleavage activity by DR-PC-DNA1 at 48°C, and with 61.3% activity recovery after 15 seconds of UV light (365 nm, 35 W) exposure. All the experiments were conducted in triplicate and error bars represent mean value +/− SD (n = 3). Source data are provided as a Source Data file.

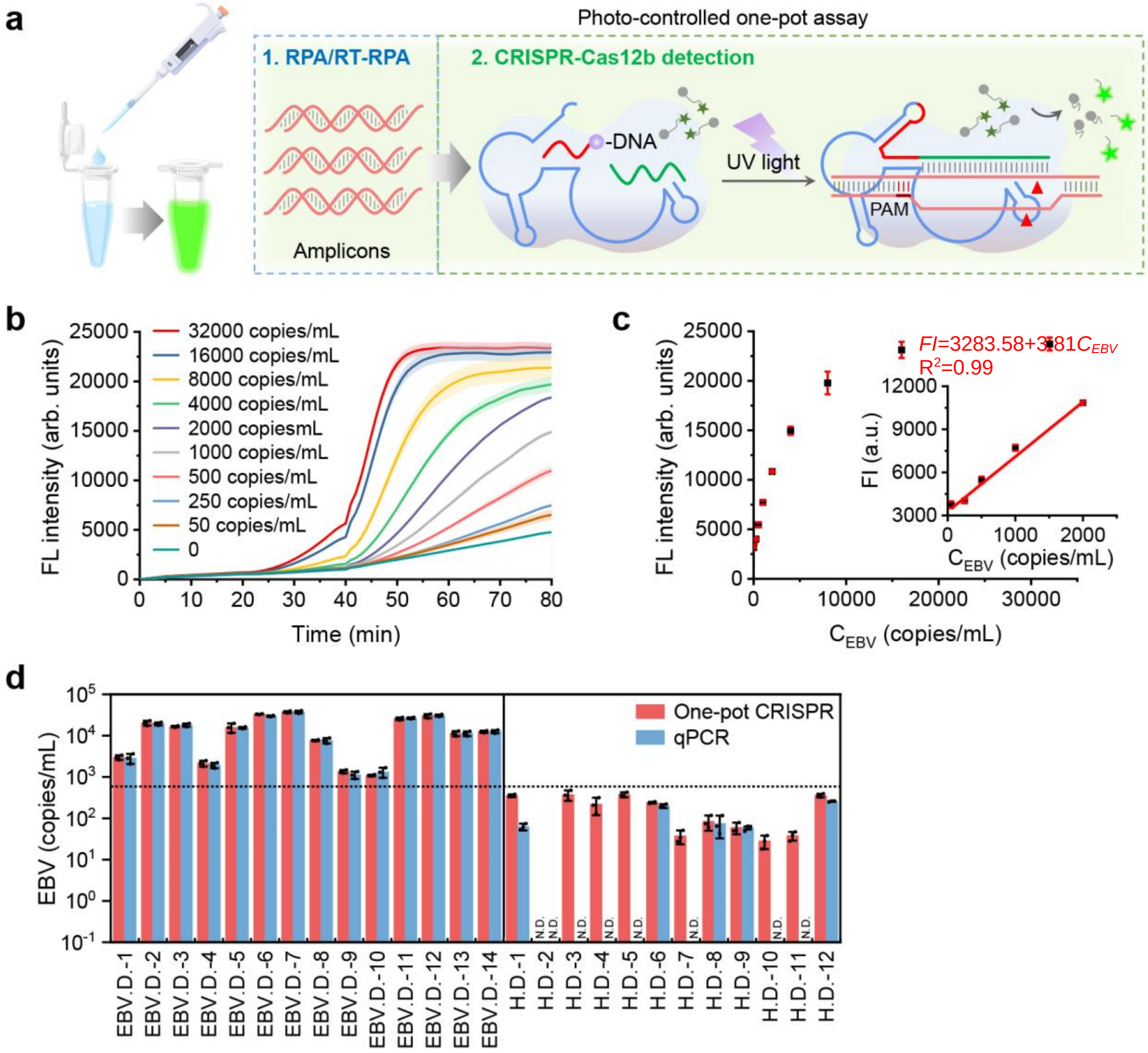

Supplementary Fig. 19. Photo-controlled one-pot RPA-CRISPR-Cas12b system for Clinical EBV sample analysis using DR-PC-DNA (a) Diagram illustrating the combination of the tracrRNA+DR+Spacer strategy with RPA technology, incorporating DR-PC-DNA, for one-pot RPA-CRISPR-Cas12b detection of nucleic acid targets. (b) Real-time fluorescence graphs for the detection of different concentrations of EBV standard using the one-pot tracrRNA+DR-PC-DNA+Spacer assisted RPA-CRISPR-Cas12b method. Data represent the mean and standard deviation of three technical replicates. (c) Linear fit graphs for the detection of EBV. (d) Bar chart comparing the analysis of traditional qPCR methods with the currently developed split sgRNA-assisted one-pot RPA-CRISPR-Cas12b methods for different clinic blood samples infected with or without EBV. All the experiments were conducted in triplicate and error bars represent mean value +/− SD (n = 3). N.D. indicates not detected. Source data are provided as a Source Data file. This Supplementary Fig. 19a is adapted from Wang, J., Zhang, W., Li, W., Xie, Q., Zang, Z. and Liu, C. Enhancement of

CRISPR-Cas12a system through universal circular RNA design. *Cell Rep. Methods* 5, 101076 (2025),[2] licensed under CC-BY 4.0 (https://creativecommons.org/licenses/by/4.0/).

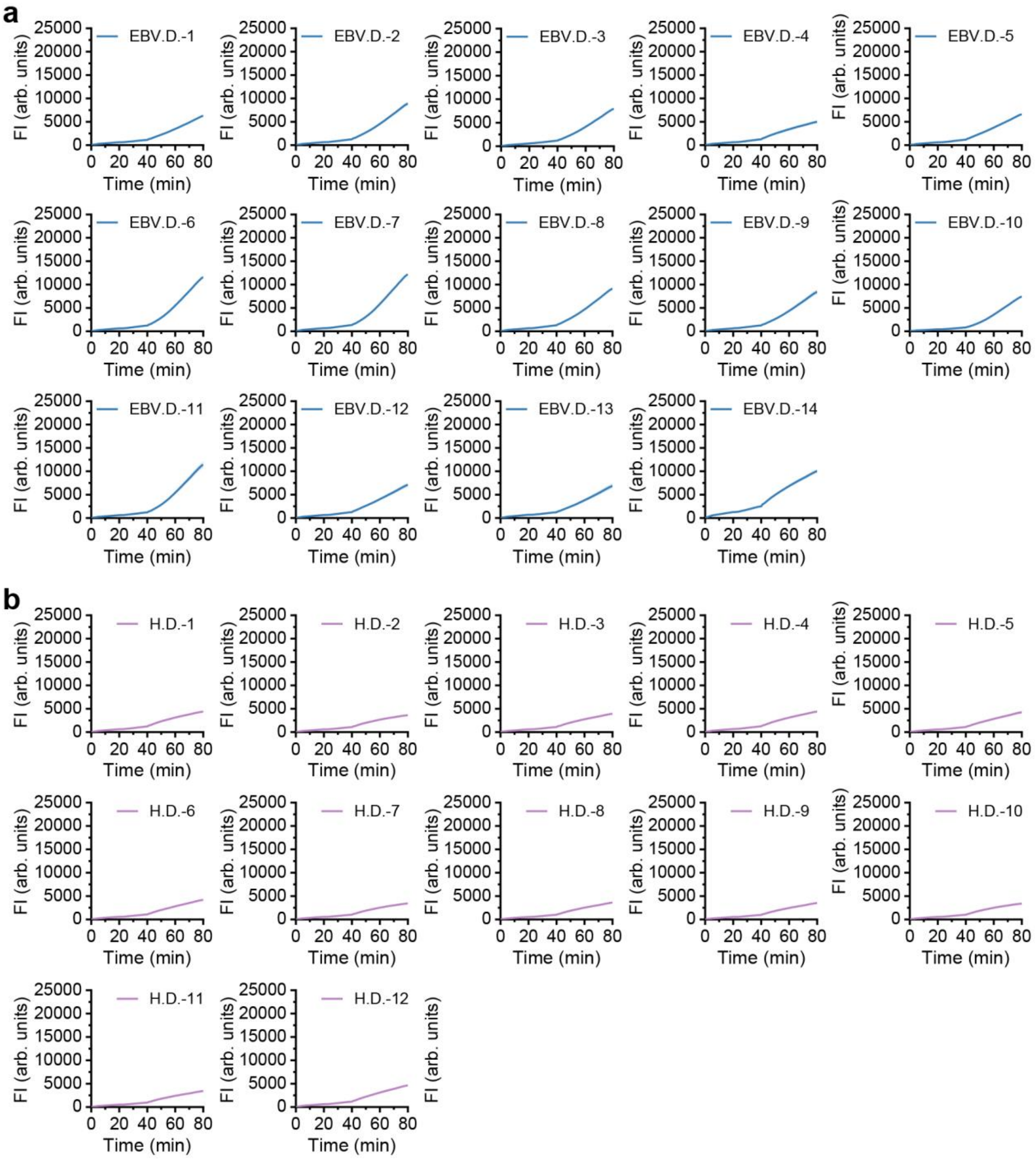

Supplementary Fig. 20. Raw fluorescence data of the clinical samples using the currently developed method. (a) Clinical plasma samples were infected with EBV. (b) Clinical plasma samples were not infected with EBV. Nucleic acids were extracted from 200 uL of plasma samples, eluted to 20 uL nuclease-free ddH2O, and 2 uL of each sample was added to the one-pot detection system. The volume was then filled up to 20

uL with nuclease-free ddH_2O. After 20 min of RPA at 37°C, the samples were exposed to UV light for 15 seconds, and fluorescence variations were monitored at 37°C for 20 min and 48°C for 40 min. All the experiments were conducted in triplicate and error bars represent mean value +/− SD (n = 3). Source data are provided as a Source Data file.

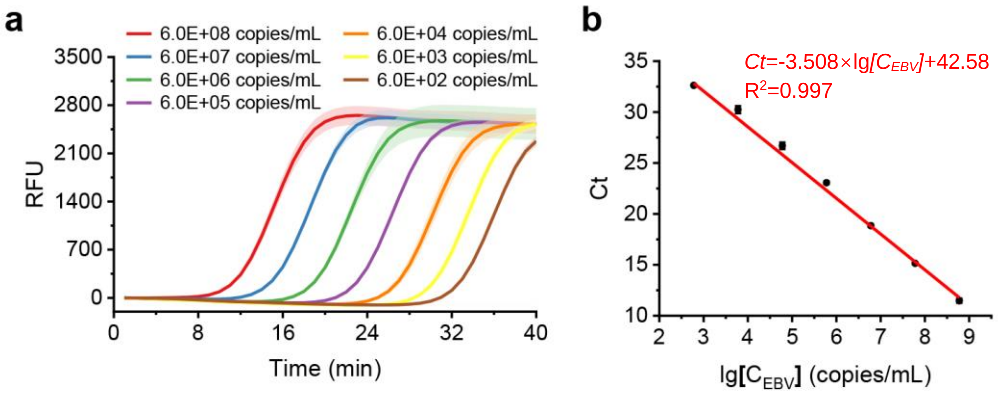

Supplementary Fig. 21. Standard curve of real-time quantitative PCR. (a) Real-time fluorescence graphs for the detection of different concentrations of EBV standard using the traditional qPCR method. Data represent the mean and standard deviation of three technical replicates. (b) Corresponding linear fit graphs. All the experiments were conducted in triplicate and error bars represent mean value +/− SD (n = 3). Source data are provided as a Source Data file.

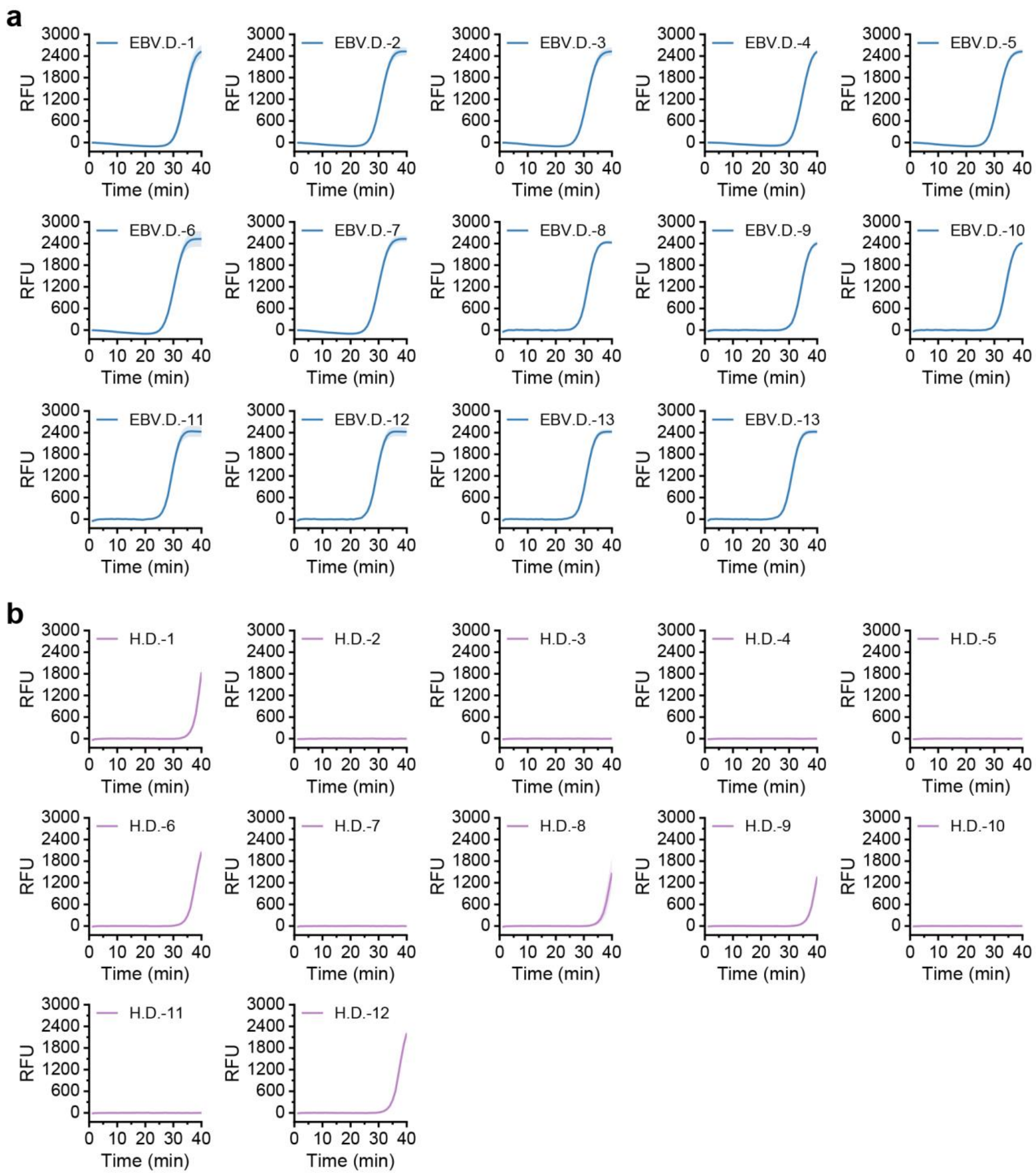

Supplementary Fig. 22. Real-time quantitative PCR for clinical EBV sample detection. Raw fluorescence data were recorded for the detection of clinical plasma samples using the traditional qPCR method, for comparison with Supplementary Fig. 13, whether the samples were clinically diagnosed as infected (a) or not (b) with EBV. All the experiments were conducted in triplicate and error bars represent mean value +/− SD (n = 3). Source data are provided as a Source Data file.

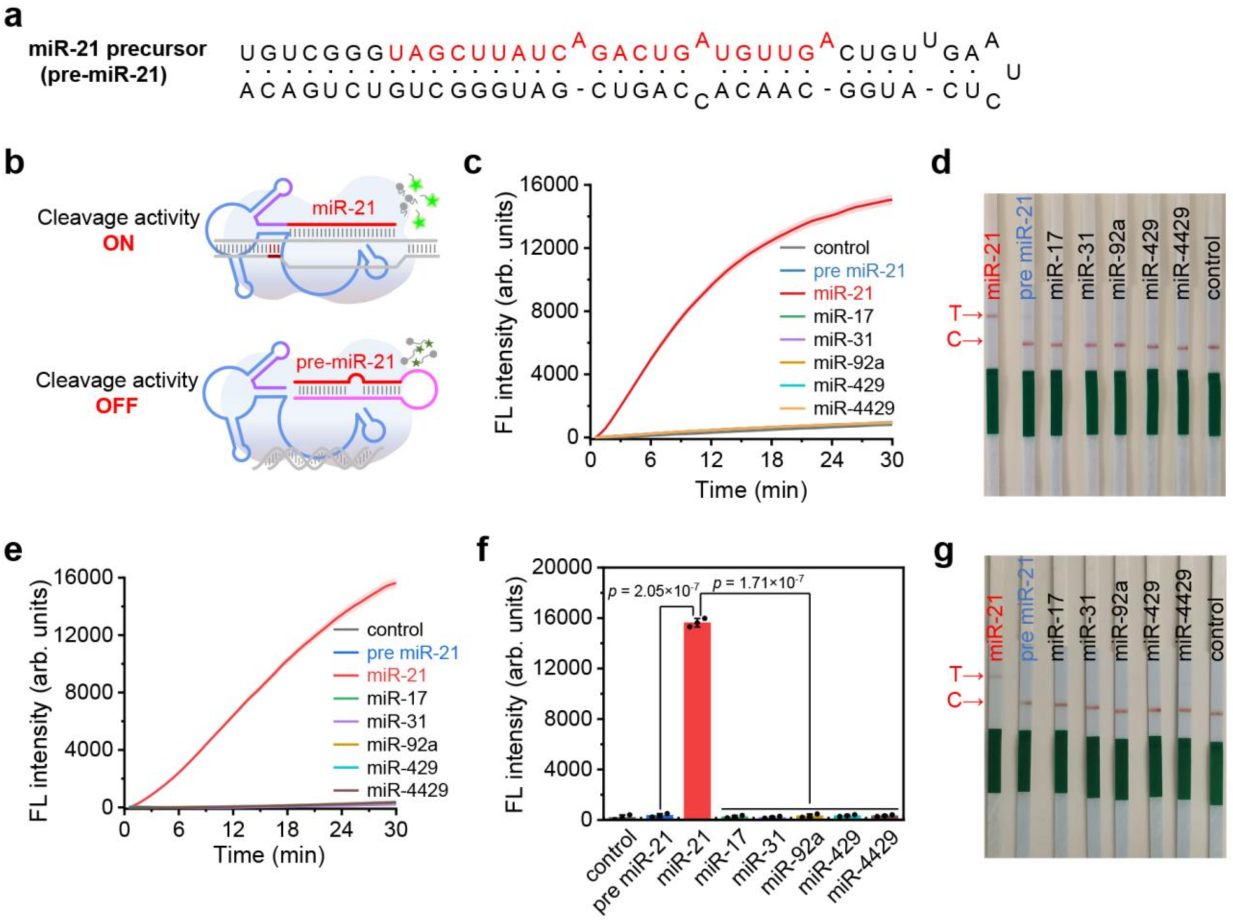

Supplementary Fig. 23. Amplification-free CRISPR-Cas12b system for microRNA detection. (a) Sequence and secondary structure of the miR-21 precursor, with the mature miR-21 sequence highlighted in red. (b) Schematic illustration of the split sgRNA strategy for detecting miR-21 and its precursor. In the miR-21 precursor, the mature miR-21 sequence is partially occluded by its complementary strand, preventing its binding to Cas12b and thereby inhibiting the activation of Cas12b's trans-cleavage activity. This design highlights the specificity of the split sgRNA approach in distinguishing between the mature miR-21 and its precursor. (c) Real-time quantitative fluorescence raw data validating the selective detection of different microRNAs using the split strategy (tracrDR) in conjunction with a DNA activator specific to miR-21. Notably, other microRNAs and the precursor of miR-21 do not produce fluorescence. (d) Detection of miR-21 utilizing commercial test strips with the split strategy (tracrDR). The photograph displays the test strip results, with the T line indicating the presence of miR-21 and the C line serving as a control. (e) Real-time quantitative fluorescence raw data validating the selective detection of different microRNAs using the split strategy (tracrRNA+DR) paired with a DNA activator specific to miR-21, again highlighting that other microRNAs and the precursor of miR-21 yield no fluorescence. (f) Bar graph analysis corresponding to panel (e). (g) Detection of miR-21 using commercial test strips with the split strategy (tracrRNA+DR). The

photograph demonstrates the test strip results, where the T line denotes the presence of miR-21 and the C line acts as a control. All the experiments were conducted in triplicate and error bars represent mean value +/− SD (n = 3). Source data are provided as a Source Data file. This Supplementary Fig. 23b is adapted from Wang, J., Zhang, W., Li, W., Xie, Q., Zang, Z. and Liu, C. Enhancement of CRISPR-Cas12a system through universal circular RNA design. *Cell Rep. Methods* 5, 101076 (2025),[2] licensed under CC-BY 4.0 (https://creativecommons.org/licenses/by/4.0/).

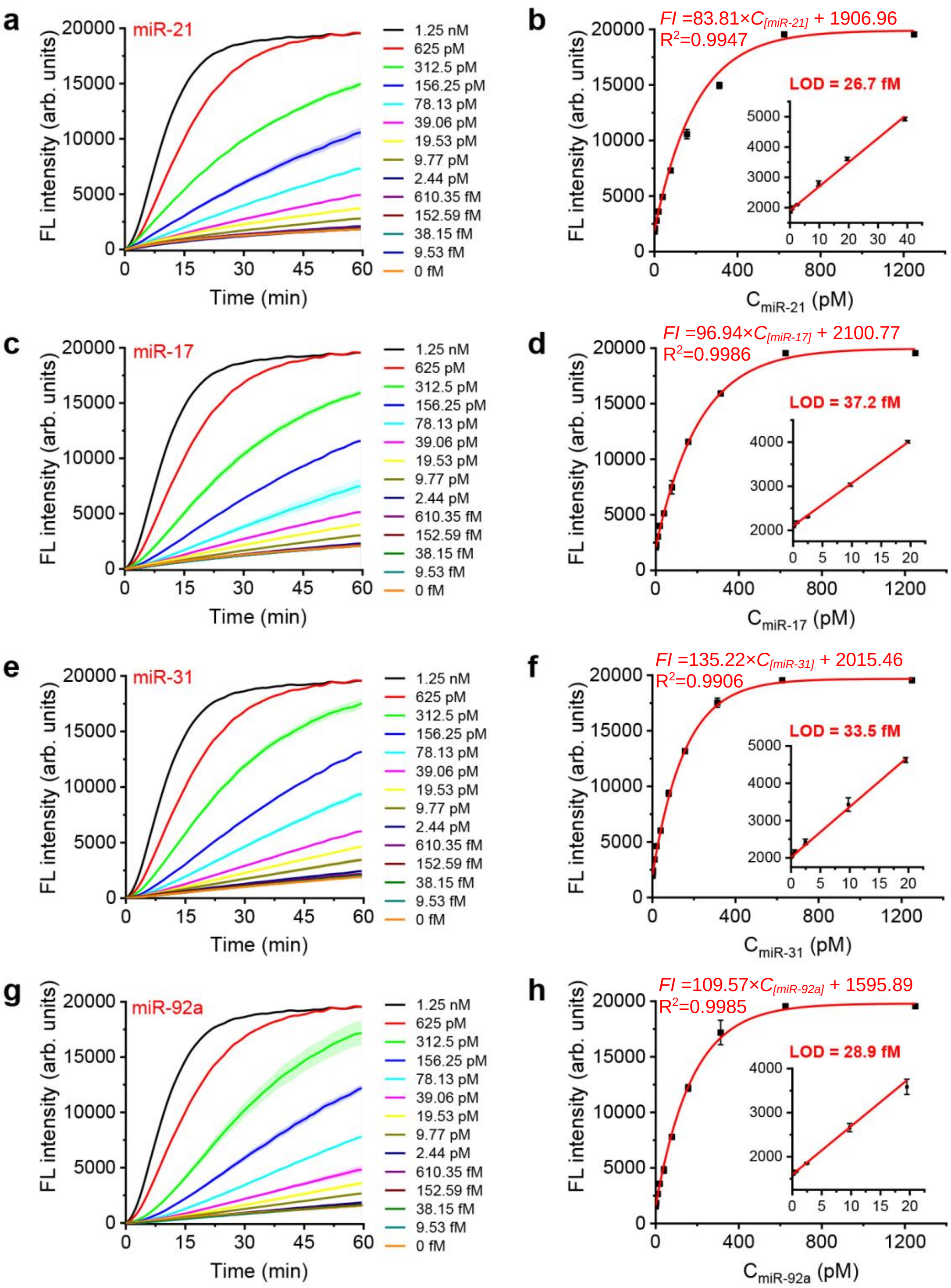

Supplementary Fig. 24. Standard curve of microRNA detection using the currently developed method. (a) Real-time fluorescence intensity curves illustrating the performance of the currently developed split strategy (tracrDR) paired with a miR-21 DNA activator at different concentrations of miR-21 standards. (b) Data from panel (a) are presented in a curve graph format along with linear regression analysis and limits

of detection. (c) Real-time fluorescence intensity curves showing the performance of the split strategy (tracrDR) with a miR-17 DNA activator at different concentrations of miR-17 standards. (d) Data from panel (c) are shown in a curve graph format along with linear regression analysis and limits of detection. (e) Real-time fluorescence intensity curves related to the currently developed split strategy (tracrDR) paired with a miR-31 DNA activator, measured over different concentrations of miR-31 standards. (f) Data from panel (e) are graphically depicted, including linear regression analysis and limits of detection. (g) Real-time fluorescence intensity curves for the split strategy (tracrDR) paired with a miR-92a DNA activator at different concentrations of miR-92a standards. (h) Data from panel (g) are shown graphically with linear regression analysis and limits of detection. All the experiments were conducted in triplicate and error bars represent mean value +/− SD (n = 3). Source data are provided as a Source Data file.

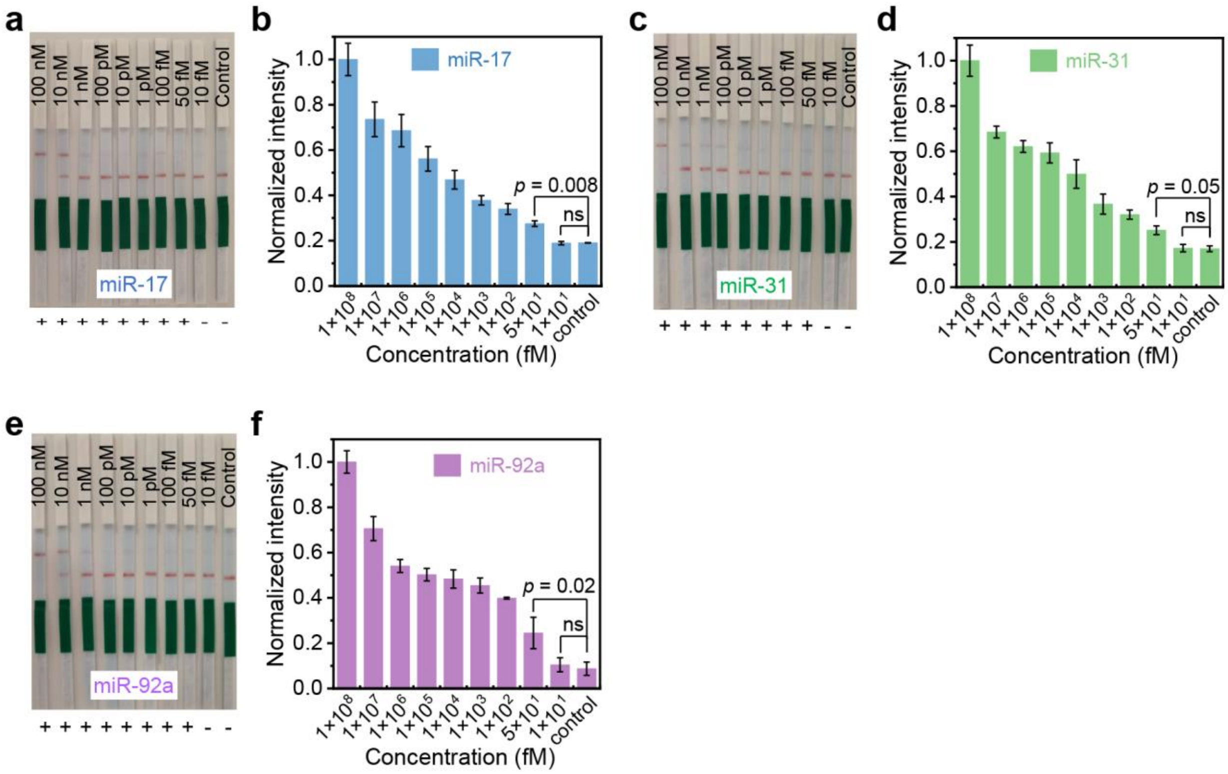

Supplementary Fig. 25. Commercial test strips detection of different microRNA with various concentrations. The photograph displays the test strip results, where the T line signifies the presence of miR-17 (a), miR-31 (c), miR-92a (e) and the C line serves as a control. Schematic representation of miR-17 (b), miR-31 (d), miR-92a (f) of ImageJ quantitative analysis applied to the results shown in test strip results. All the experiments were conducted in triplicate and error bars represent mean value +/− SD (n = 3).

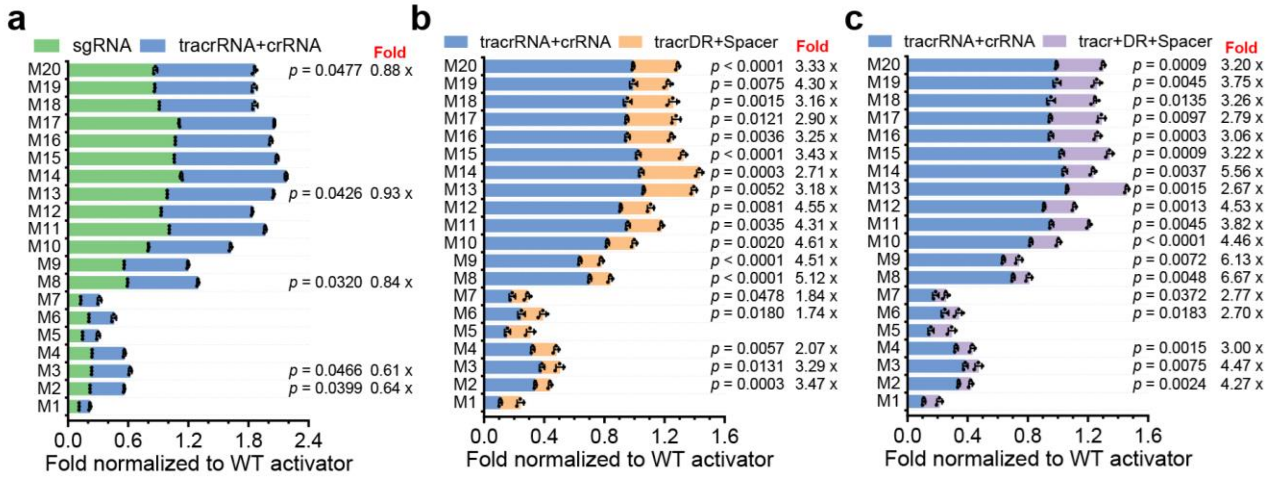

Supplementary Fig. 26. Comparison of fluorescence readings for four sgRNA types when detecting wild-type and single base mutations (M1-20). (a) Comparison of fluorescence readings between full-length sgRNA and split sgRNA (tracrRNA+crRNA) in detecting wild-type and single base mutations (M1-20). The y-axis corresponds to the mutation position, which aligns with the data presented in panel (Figure 4e), while the x-axis displays the ratio of fluorescence values derived from the mutated sequences relative to the wild type. (b) Comparison of fluorescence readings for tracrRNA+crRNA versus tracrDR+Spacer under conditions of wild-type and single base mutations (M1-20). Again, the y-axis represents the mutation position in line with panel (Figure 4e), with the x-axis indicating the fluorescence value ratios from the mutated sequences against the wild type. (c) Comparison of fluorescence readings for tracrRNA+crRNA versus tracrRNA+DR+Spacer, similarly evaluating wild-type and single base mutations (M1-20). The y-axis denotes the mutation position, consistent with panel (Figure 4e), while the x-axis shows the ratio of fluorescence values from mutated sequences compared to the wild type. The green bars indicate full-length sgRNA, blue bars represent full-length tracrRNA+crRNA, brown bars denote the tracrDR+Spacer configuration, and pink bars signify the tracrRNA+DR+Spacer configuration. *P* value denote statistically significant differences between the groups, with the accompanying numbers reflecting the fold change. All the experiments were conducted in triplicate and error bars represent mean value +/− SD (n = 3), and statistical analysis was conducted using a two-tailed t-test. Source data are provided as a Source Data file.

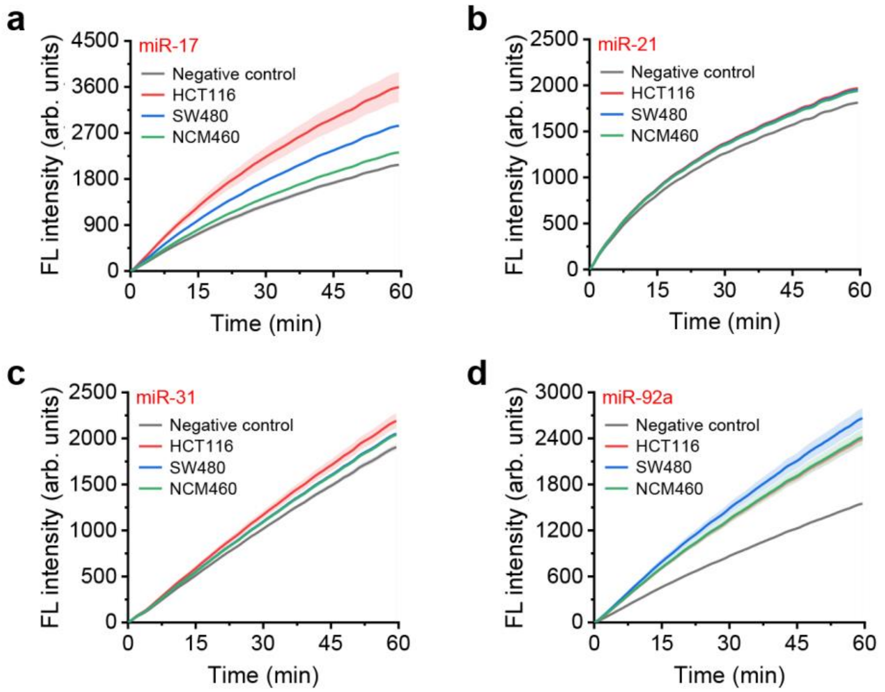

Supplementary Fig. 27. MicroRNA detection of the developed split strategy (tracrDR) across HCT116, SW480, and NCM460 cell lines. Real-time fluorescence intensity curves illustrating the performance of the developed split strategy (tracrDR) paired with (a) miR-17 DNA activator in detecting miR-17, (b) miR-21 DNA activator in detecting miR-21, (c) miR-31 DNA activator in detecting miR-31, (d) miR-92a DNA activator in detecting miR-92a. All the experiments were conducted in triplicate and error bars represent mean value +/− SD (n = 3). Source data are provided as a Source Data file.

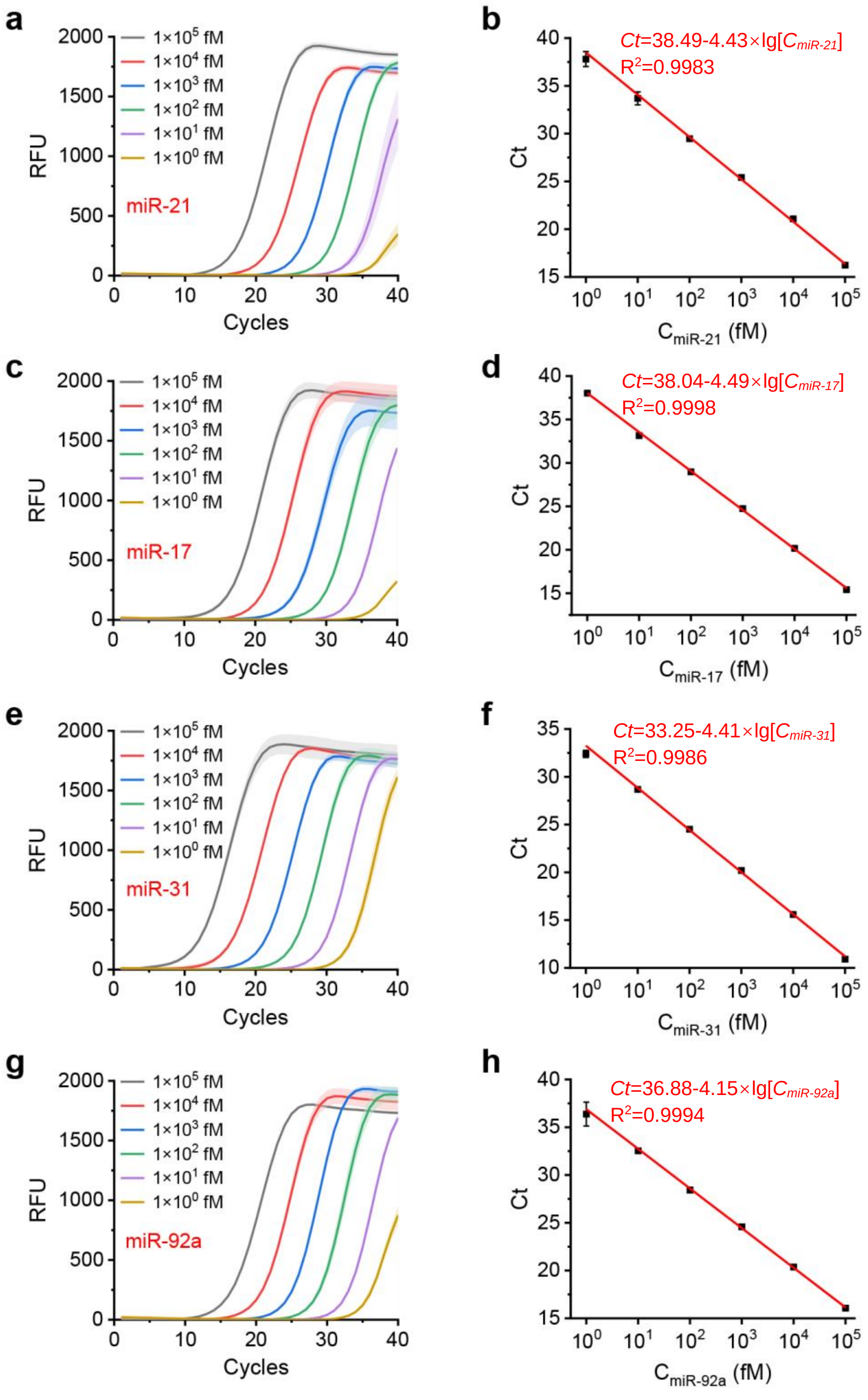

Supplementary Fig. 28. Real-time fluorescence curves obtained from conventional RT-qPCR for varying concentrations of different microRNA standards. (a) miR-21, (c) miR-17, (e) miR-31, and (g) miR-92a, along with their corresponding linear regression analyses: (b) miR-21, (d) miR-17, (f) miR-31, and (h) miR-

92a. Error bars represent standard deviation (n=3). All the experiments were conducted in triplicate and error bars represent mean value +/− SD (n = 3). Source data are provided as a Source Data file.

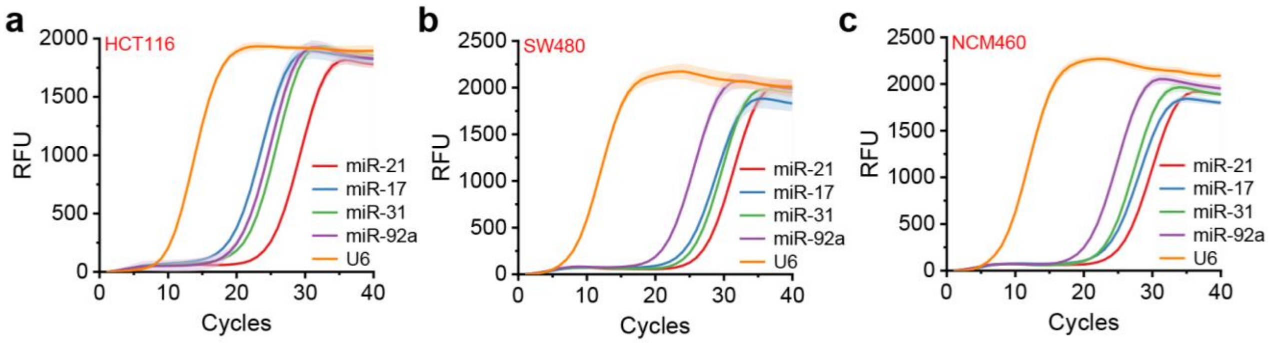

Supplementary Fig. 29. Real-time fluorescence curves of microRNA detection in cell lines. Raw data from conventional RT-qPCR for detecting miR-21, miR-17, miR-31, and miR-92a, as well as the reference gene U6, in HCT116 (a), SW480 (b), and NCM460 (c) cell lines, demonstrating the real-time fluorescence curves. All the experiments were conducted in triplicate and error bars represent mean value +/− SD (n = 3). Source data are provided as a Source Data file.

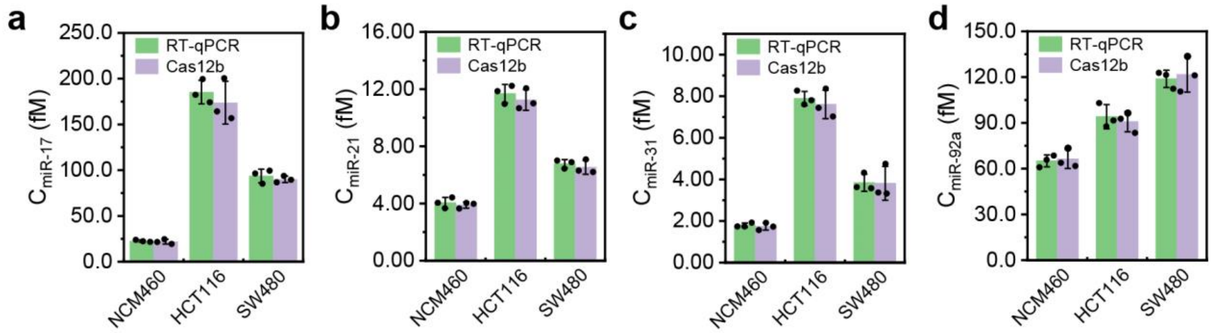

Supplementary Fig. 30. MicroRNA expression level measured by Cas12b assay (purple) and RT-qPCR (green) in cell lines. Bar graphs comparing the detection of miR-17 (a), miR-21 (b), miR-31 (c), and miR-92a (d) using the developed split strategy (tracrDR) paired with respective DNA activators in HCT116, SW480, and NCM460 cell lines against the results obtained using conventional RT-qPCR methods. All the experiments were conducted in triplicate and error bars represent mean value +/− SD (n = 3).

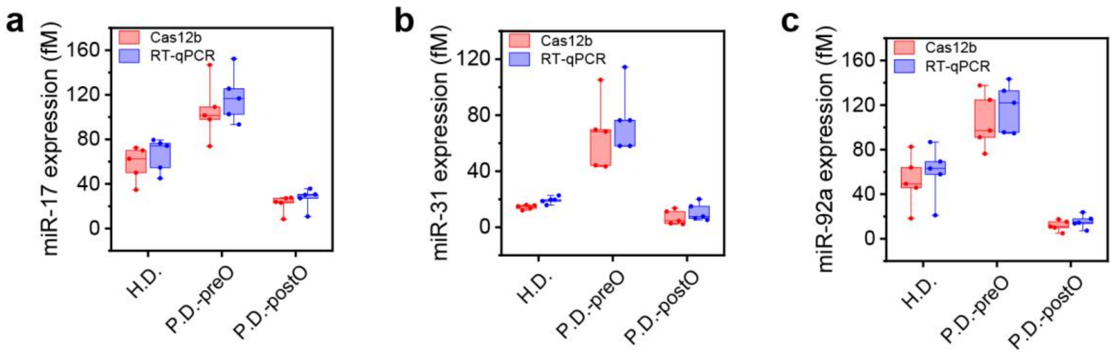

Supplementary Fig. 31. MicroRNA expression level measured by Cas12b assay (red) and RT-qPCR (blue) in colon cancer patients and healthy donors. (a) Comparison of miR-17 concentrations detected in total RNA extracted from the plasma of healthy donors (H.D.-1-5), preoperative colon cancer patients (P.D.-1-5 preO), and postoperative patients (P.D.-1-5 postO) using our novel Cas12b split sgRNA approach (tracrDR combined with miR-17-specific DNA activator) in contrast to traditional RT-qPCR methods. (b) Comparison of miR-31 concentrations in total RNA from the plasma of healthy donors, preoperative colon cancer patients, and postoperative patients using the same Cas12b split sgRNA strategy (tracrDR with miR-31-specific DNA activator) compared to traditional RT-qPCR methods. (c) Assessment of miR-92a concentrations in total RNA derived from the same plasma samples, utilizing the Cas12b split sgRNA method (tracrDR paired with miR-92a-specific DNA activator) versus traditional RT-qPCR techniques. The median expression level is depicted by the center line, the interquartile range by the box boundaries, and the whisker extrema represent the maximum and minimum values. Source data are provided as a Source Data file.

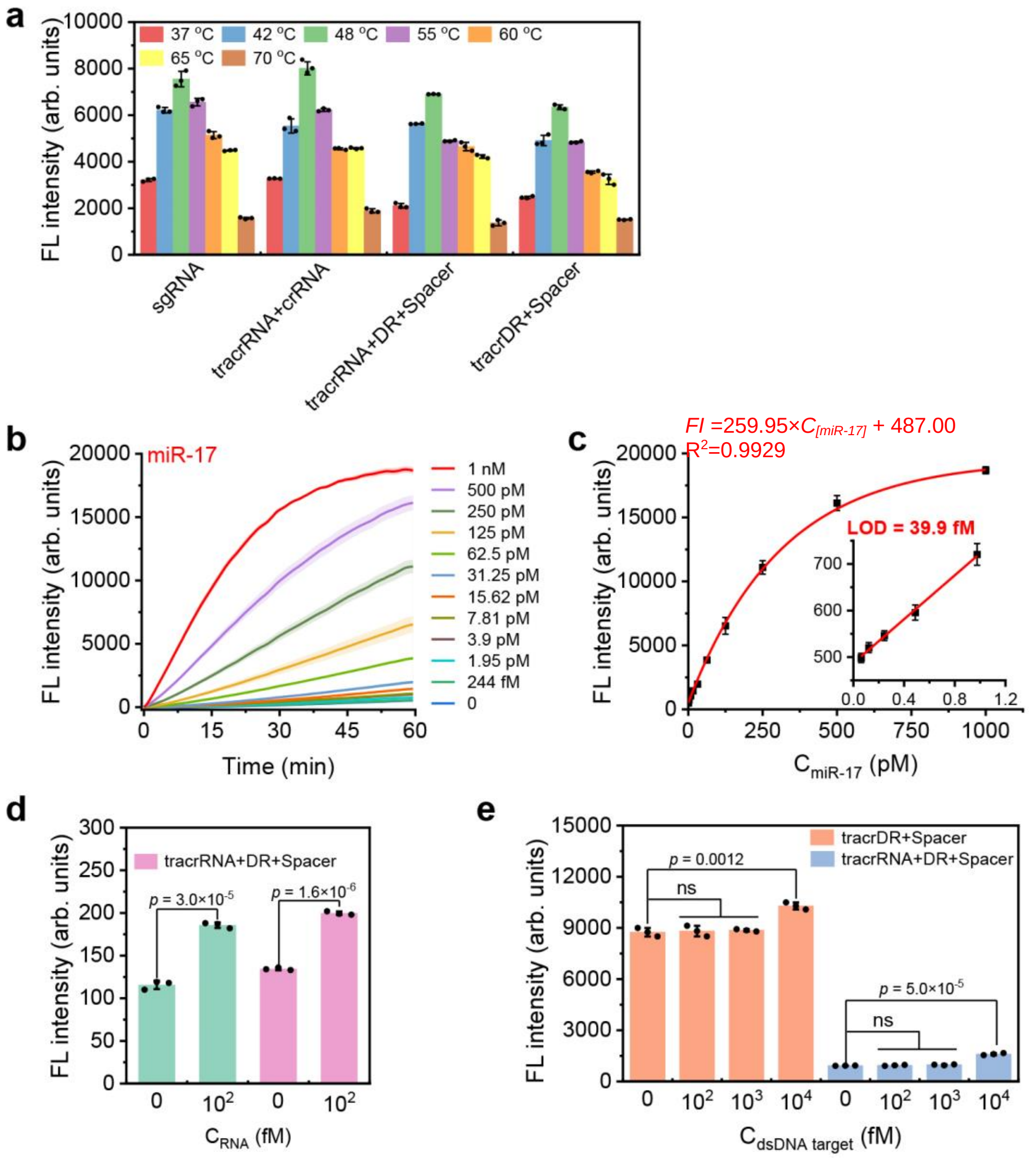

Supplementary Fig. 32. Fluorescence values of Cas12b for detecting different targets under conditions of different splits. (a) Fluorescence bar graph illustrating the trans-cleavage activity of Cas12b assisted by sgRNA, tracrRNA+crRNA, tracrDR+Spacer, and tracrRNA+DR+Spacer across a temperature range of 37–70°C. The results demonstrate that both full-length and split sgRNA exhibit peak fluorescence intensity at 48°C, confirming the robust temperature tolerance of Cas12b, including its stability at elevated temperatures. (b) Real-time fluorescence intensity curves depicting the performance of the split sgRNA strategy (tracrDR) with a single-stranded DNA activator (miR-17 DNA activator, which is distinct from the double-stranded DNA activator used in Supplementary Fig. 24c) at varying concentrations of miR-17 standards. (c) Data from panel (b) presented as a curve graph with linear regression analysis and limits of detection. The detection limits are comparable to those in Supplementary Fig. 24c, indicating that the use of either single-stranded or double-stranded DNA activators does not significantly affect the sensitivity of

microRNA detection. (d) Comparative analysis of the detection performance of 500 nM tracrDR or tracrRNA+DR in complex with Cas12b and 500 nM double-stranded target DNA (as the Spacer activator) across a range of Spacer concentrations. The system achieves a detection limit as low as 10^2 fM. (e) Comparative analysis of the detection performance of 500 nM tracrDR+Spacer or tracrRNA+DR+Spacer in complex with Cas12b across a range of double-stranded target DNA concentrations. The system achieves a detection limit as low as 10^4 fM. All the experiments were conducted in triplicate and error bars represent mean value +/− SD (n = 3). Source data are provided as a Source Data file.

Supplementary Reference

1. Liu, Y. et al. Bisulfite-free direct detection of 5-methylcytosine and 5-hydroxymethylcytosine at base resolution. *Nat. Biotechnol.* **37**, 424-429 (2019).
2. Wang, J. et al. Enhancement of CRISPR-Cas12a system through universal circular RNA design. *Cell Rep. Methods* **5**, 101076 (2025).
