# Supplementary material for: Regulating cleavage activity and enabling microRNA detection with split sgRNA in Cas12b
Source: Nat Commun. 2025 Jul 10;16:6392. doi: 10.1038/s41467-025-61748-4 (PMC12246129; doi:10.1038/s41467-025-61748-4)
Supplement: Supplementary file 2 — Reporting Summary [file 41467_2025_61748_MOESM2_ESM.pdf]

nature portfolio

Corresponding author(s): Chaoxing Liu

Last updated by author(s): Jun 6, 2025

Reporting Summary

Nature Portfolio wishes to improve the reproducibility of the work that we publish. This form provides structure for consistency and transparency in reporting. For further information on Nature Portfolio policies, see our Editorial Policies and the Editorial Policy Checklist.

Statistics

For all statistical analyses, confirm that the following items are present in the figure legend, table legend, main text, or Methods section.

n/a	Confirmed	
☐	☒	The exact sample size (*n*) for each experimental group/condition, given as a discrete number and unit of measurement
☐	☒	A statement on whether measurements were taken from distinct samples or whether the same sample was measured repeatedly
☐	☒	The statistical test(s) used AND whether they are one- or two-sided<br>*Only common tests should be described solely by name; describe more complex techniques in the Methods section.*
☒	☐	A description of all covariates tested
☒	☐	A description of any assumptions or corrections, such as tests of normality and adjustment for multiple comparisons
☐	☒	A full description of the statistical parameters including central tendency (e.g. means) or other basic estimates (e.g. regression coefficient) AND variation (e.g. standard deviation) or associated estimates of uncertainty (e.g. confidence intervals)
☐	☒	For null hypothesis testing, the test statistic (e.g. *F*, *t*, *r*) with confidence intervals, effect sizes, degrees of freedom and *P* value noted<br>*Give P values as exact values whenever suitable.*
☒	☐	For Bayesian analysis, information on the choice of priors and Markov chain Monte Carlo settings
☒	☐	For hierarchical and complex designs, identification of the appropriate level for tests and full reporting of outcomes
☒	☐	Estimates of effect sizes (e.g. Cohen's *d*, Pearson's *r*), indicating how they were calculated

Our web collection on statistics for biologists contains articles on many of the points above.

Software and code

Policy information about availability of computer code

Data collection	No specific software was used to collect data.
Data analysis	Data are shown as mean value +/− SD (n = 3) of biologically independent samples. Data analysis and processing were performed using Origin software, ImageJ software or GraphPad Prism 9.0. The three-dimensional structures of Cas12b complexes with full-length or split sgRNA configurations were predicted using AlphaFold3. Molecular dynamics (MD) simulations of Cas12b in complex with either full-length or split sgRNA were conducted using GROMACS 2024.5, with the amber14sb_OL15 force field applied to ensure accurate representation of molecular interactions and dynamics. Trajectory files generated from the MD simulations were systematically analyzed using GROMACS tools to extract critical structural and dynamic parameters. For comprehensive data visualization and interpretation, PyMol 3.0.4 and Python 3.12.2 were employed, enabling detailed structural analysis and dynamic behavior representation of the Cas12b-sgRNA complexes.

For manuscripts utilizing custom algorithms or software that are central to the research but not yet described in published literature, software must be made available to editors and reviewers. We strongly encourage code deposition in a community repository (e.g. GitHub). See the Nature Portfolio guidelines for submitting code & software for further information.

Data

Policy information about availability of data

All manuscripts must include a data availability statement. This statement should provide the following information, where applicable:

- Accession codes, unique identifiers, or web links for publicly available datasets
- A description of any restrictions on data availability
- For clinical datasets or third party data, please ensure that the statement adheres to our policy

All data supporting this study are available within the article, Supplementary Files, and dedicated Source Data files. Structural coordinates referenced in this work are deposited under PDB ID: 5U34 (https://www.rcsb.org/structure/5U34). Source data are provided with this paper.

Research involving human participants, their data, or biological material

Policy information about studies with human participants or human data. See also policy information about sex, gender (identity/presentation), and sexual orientation and race, ethnicity and racism.

Reporting on sex and gender	All human clinical samples were collected in a randomized manner without further stratification based on gender. The clinical samples in this proof-of-concept study were collected randomly without intentional stratification by sex or gender. As this work primarily focuses on establishing technical feasibility for detecting trace nucleic acid biomarkers using our novel split sgRNA design, the preliminary analysis did not include sex/gender comparisons.
Reporting on race, ethnicity, or other socially relevant groupings	N/A
Population characteristics	N/A
Recruitment	N/A
Ethics oversight	Human blood samples were collected and provided by the Seventh Affiliated Hospital, Sun Yat-sen University with protocols approved by the ethics committee at the Seventh Affiliated Hospital, Sun Yat-sen University (KY-2024-192-01, KY-2024-384-02, KY-2024-009-02 and KY-2023-117-01). All patients in this study signed an informed consent form.

Note that full information on the approval of the study protocol must also be provided in the manuscript.

Field-specific reporting

Please select the one below that is the best fit for your research. If you are not sure, read the appropriate sections before making your selection.

☒ Life sciences ☐ Behavioural & social sciences ☐ Ecological, evolutionary & environmental sciences

For a reference copy of the document with all sections, see nature.com/documents/nr-reporting-summary-flat.pdf

Life sciences study design

All studies must disclose on these points even when the disclosure is negative.

Sample size	The sample size was determined based on previous publications related to this study, typically involving at least three replicates.
Data exclusions	No data were excluded.
Replication	All experiments were conducted with three replicates unless otherwise specified, and all were successfully replicated.
Randomization	Human clinical samples were collected randomly without stratification by gender or other factors. For proof-of-concept validation of the methods described in the manuscript, 14 plasma samples from EBV-infected patients and 12 samples from healthy donors were collected. Additionally, plasma samples were obtained from five healthy donors and five colorectal cancer patients before and after surgery.
Blinding	The study was not designed as a double-blind experiment. The collection and analysis of clinical samples were performed as a conceptual validation, with comparisons made to established gold methods.

Reporting for specific materials, systems and methods

We require information from authors about some types of materials, experimental systems and methods used in many studies. Here, indicate whether each material, system or method listed is relevant to your study. If you are not sure if a list item applies to your research, read the appropriate section before selecting a response.

Materials & experimental systems

n/a	Involved in the study
☒	☐ Antibodies
☐	☒ Eukaryotic cell lines
☒	☐ Palaeontology and archaeology
☒	☐ Animals and other organisms
☒	☐ Clinical data
☒	☐ Dual use research of concern
☒	☐ Plants

Methods

n/a	Involved in the study
☒	☐ ChIP-seq
☒	☐ Flow cytometry
☒	☐ MRI-based neuroimaging

Eukaryotic cell lines

Policy information about cell lines and Sex and Gender in Research

Cell line source(s)	The HCT116 and SW480 cell lines were obtained from the China Center for Type Culture Collection (CCTCC, Wuhan, China). The NCM460 cell line was purchased from Wuhan Sunncell Biotechnology Co., Ltd (Wuhan, China, Cat No. SNL-519).
Authentication	All cell lines were authenticated using STR identification and confirmed to be free of contamination.
Mycoplasma contamination	All cell lines were confirmed to be free of Mycoplasma contamination.
Commonly misidentified lines (See ICLAC register)	N/A

Plants

Seed stocks	N/A
Novel plant genotypes	N/A
Authentication	N/A
